# Matriptase-dependent epidermal pre-neoplasm in zebrafish embryos caused by a combination of hypotonic stress and epithelial polarity defects

**DOI:** 10.1371/journal.pgen.1010873

**Published:** 2023-08-11

**Authors:** Julia Hatzold, Verena Nett, Stephanie Brantsch, Jin-Li Zhang, Joy Armistead, Heike Wessendorf, Rebecca Stephens, Patrick O. Humbert, Sandra Iden, Matthias Hammerschmidt

**Affiliations:** 1 Institute of Zoology–Developmental Biology, University of Cologne, Germany; 2 Cell and Developmental Biology, Center of Human and Molecular Biology (ZHMB), Saarland University, Faculty of Medicine, Homburg/Saar, Germany; 3 Center for Molecular Medicine Cologne (CMMC), University Hospital Cologne, Cologne, Germany; 4 Department of Biochemistry & Chemistry, La Trobe Institute for Molecular Science, La Trobe University, Melbourne, Victoria, Australia; 5 Department of Biochemistry and Pharmacology, University of Melbourne, Melbourne, Victoria, Australia; 6 Department of Clinical Pathology, University of Melbourne, Melbourne, Victoria, Australia; University of Pennsylvania Perelman School of Medicine, UNITED STATES

## Abstract

Aberrantly up-regulated activity of the type II transmembrane protease Matriptase-1 has been associated with the development and progression of a range of epithelial-derived carcinomas, and a variety of signaling pathways can mediate Matriptase-dependent tumorigenic events. During mammalian carcinogenesis, gain of Matriptase activity often results from imbalanced ratios between Matriptase and its cognate transmembrane inhibitor Hai1. Similarly, in zebrafish, unrestrained Matriptase activity due to loss of *hai1a* results in epidermal pre-neoplasms already during embryogenesis. Here, based on our former findings of a similar tumor-suppressive role for the Na^+^/K^+^-pump beta subunit ATP1b1a, we identify epithelial polarity defects and systemic hypotonic stress as another mode of aberrant Matriptase activation in the embryonic zebrafish epidermis *in vivo*. In this case, however, a different oncogenic pathway is activated which contains PI3K, AKT and NFkB, rather than EGFR and PLD (as in *hai1a* mutants). Strikingly, epidermal pre-neoplasm is only induced when epithelial polarity defects in keratinocytes (leading to disturbed Matriptase subcellular localization) occur in combination with systemic hypotonic stress (leading to increased proteolytic activity of Matriptase). A similar combinatorial effect of hypotonicity and loss of epithelial polarity was also obtained for the activity levels of Matriptase-1 in human MCF-10A epithelial breast cells. Together, this is in line with the multi-factor concept of carcinogenesis, with the notion that such factors can even branch off from one and the same initiator (here ATP1a1b) and can converge again at the level of one and the same mediator (here Matriptase). In sum, our data point to tonicity and epithelial cell polarity as evolutionarily conserved regulators of Matriptase activity that upon de-regulation can constitute an alternative mode of Matriptase-dependent carcinogenesis *in vivo*.

## Author summary

Deciphering the mechanisms of carcinogenesis is pivotal not only for the treatment of the disease, but also for identifying potential risk factors. Model systems can help to achieve this goal. Work with zebrafish has identified several evolutionarily conserved factors driving pre-neoplastic events in the epidermis already at embryonic stages. This study focuses on the oncogene ST14, encoding the type II transmembrane protease Matriptase-1 that upon hyperactivation—as for instance due to genetic loss of its cognate transmembrane inhibitor Hai1—can drive different carcinomas. Here, we identify two additional conditions promoting oncogenic Matriptase-1 hyperactivation: hypotonic stress and compromised epithelial cell polarity. Of note, these two conditions need to occur together for Matriptase-dependent induction of pre-neoplasms in the embryonic zebrafish epidermis, in line with the multi-factor concept of carcinogenesis. Hypotonicity increases the proteolytic activity of Matriptase, while epithelial polarity defects lead to a subcellular mis-localization of Matriptase in outer epidermal cells, bringing more of it into close contact to underlying basal cells and thus inducing oncogenic pathways in them. Together, our work identifies novel modes of oncogenic Matriptase hyperactivation and function *in vivo*, originating from cells of the tumor environment, rather than from the tumor cells themselves.

## Introduction

The genetic basis of carcinogenesis is highly complex and variable. Depending on context and tissue, dysregulated oncogenes can lead to different outcomes, sometimes even involving different downstream signaling pathways. For example, the type II serine transmembrane protease Matriptase-1, also named Suppressor of tumorigenicity 14 (St14), has been reported as a potential oncogene in a wide range of epithelial derived cancers, including breast cancer, colorectal cancer, and squamous cell carcinomas [1,2,3]. Aberrant Matriptase activity is associated with tumor initiation as well as progression and metastasis. Underlying pathways are diverse and can include different growth factors and cell surface receptors such as Hepatocyte growth factor (HGF) and its receptor c-Met, Macrophage-stimulating protein (MST) and its receptor Ron, and/or the proteolysis-activated receptor Par2, as well as different intracellular signal transducers such as phosphatidylinositol-3-kinase (PI3K), MAP-kinase or the transcription factor NFkB [1,4,5,6]. Matriptase is synthesized as a zymogen and trafficked to the cell surface, where it undergoes tightly controlled auto-activation [7]. In cell culture systems and biochemical in *vitro* assays, respectively, this auto-activation has been shown to be influenced by the subcellular localization of Matriptase [8,9] and by pericellular environmental conditions, with highest activation levels at mildly acidic pH and low ionic strength [10,11,12]. In healthy tissue, Matriptase activity is usually restrained by its cognate inhibitors, the Kunitz-type serine protease inhibitors Hai-1 and Hai-2, also named Spint1 and Spint2, while an imbalanced ratio of Matriptase and its inhibitors results in Matriptase dysregulation and pathology [13]. However, other modes of pathological / oncogenic Matriptase hyperactivation are feasible as well, but, at least with respect to their *in vivo* relevance, little understood.

In recent years, not only adult but also embryonic zebrafish have evolved as animal models of carcinogenesis [14,15,16,17,18], in line with the concept of oncofetal reprogramming in tumor development and progression [19]. Several zebrafish mutants have been identified that display early stages of carcinogenesis in the bi-layered embryonic epidermis, characterized by keratinocyte hyper-proliferation and aggregate formation, as well as partial or complete epithelial-mesenchymal transitions (EMTs) of keratinocytes. Among them are loss-of-function mutants in *hai1a* [20,21,22,23,24,25], in which epidermal pre-neoplasm but also concomitant tumor-suppressive processes occur as direct consequences of increased Matriptase-1 activity, mediated by the Protease-activated receptor 2b (Par2b) and a linear EGFR (Epidermal growth factor receptor)–PLD (Phospholipase D) signal transduction pathway [20].

Even more advanced epidermal carcinogenesis, including the invasion of zebrafish embryo internal tissues by transformed basal keratinocytes, is caused by loss-of-function mutations in *atp1b1a*, encoding a β-subunit of a Na^+^/K^+^-ATPase [26,27]. In this case, carcinogenesis is initiated by the combination of two effects resulting from the loss of ATP1b1a, which only in conjunction lead to the activation of an oncogenic PI3K - AKT (protein kinase B)—mTORC1—NFkB pathway in basal keratinocytes [26]: first, the loss of epithelial polarity in keratinocytes of the outer epidermal layer, also called peridermal cells, in line with the formerly described function of ATPb1 as an epithelial polarity regulator in *Drosophila* embryos [28], and second, systemic hypotonic stress, most likely due to impaired kidney function, in line with the known role of ATPb1 as an ion pump component and osmoregulator [29]. Accordingly, epidermal malignancy can be prevented both by re-introducing wild-type ATP1b1a into outer peridermal cells to reconstitute epithelial polarity, as well as by incubating embryos in isotonic rather than their natural hypotonic medium [26].

Epithelial polarity is known to play important roles in tissue architecture and homeostasis [30,31], and loss of epithelial polarity has long been associated with EMT and tumorigenesis. Polarity proteins like Par3, Lethal giant larvae (Lgl) or Scribble have been described as tumor suppressors in several contexts [32,33,34,35,36,37], Lgl2 even in the context of the embryonic zebrafish epidermis [38,39]. In comparison, rather little had been known about hypotonic stress as a potential oncogenic factor, with only few indirect indications [40,41].

Here, we set out to test the impact of hypotonicity on other zebrafish mutants with embryonic preneoplastic epidermal aggregates [21,22,42] and found that, as seen for *atp1b1a* mutants [26], isotonic medium alleviates the epidermal defects of *hai1a* mutants, similar to the formerly described effect of genetic inactivation of Matriptase-1 in *hai1a* mutants [20,21]. Furthermore and surprisingly, as *hai1a* mutants [20,21], also *atp1b1a* mutants display a normalization of their epidermal defects upon genetic inactivation of Matriptase-1. These data identify Matriptase-1 as the missing link and thus far unknown factor at the convergence point of the epithelial polarity defects in outer keratinocytes and the systemic hypotonic stress of *atp1b1a* mutants, activating the oncogenic PI3K-AKT-mTORC1-NFkB pathway in basal keratinocytes. We further show that the polarity defects in outer keratinocytes of *atp1b1a* mutants lead to a mis-localization of Matriptase-1 from lateral to basal domains facing the underlying basal keratinocytes, while hypotonic stress increases the enzymatic activity of Matriptase to proteolytically cleave Par2b, thereby activating the oncogenic pathway in basal keratinocytes. These studies do not only shed further light onto the tumor-suppressive mechanisms of ATP1b1a, but, together with additional data obtained in mammalian epithelial cell culture and other embryonic zebrafish systems, also identify hypotonicity and epithelial polarity defects as novel regulators of Matriptase-1 activity and its oncogenic potential.

## Results

### Isotonicity and loss of Matriptase activity alleviate epidermal hyperplasia of *hai1a and atp1b1a*, but not of *epcam* and *clint1a* mutants

**Fig 1:**
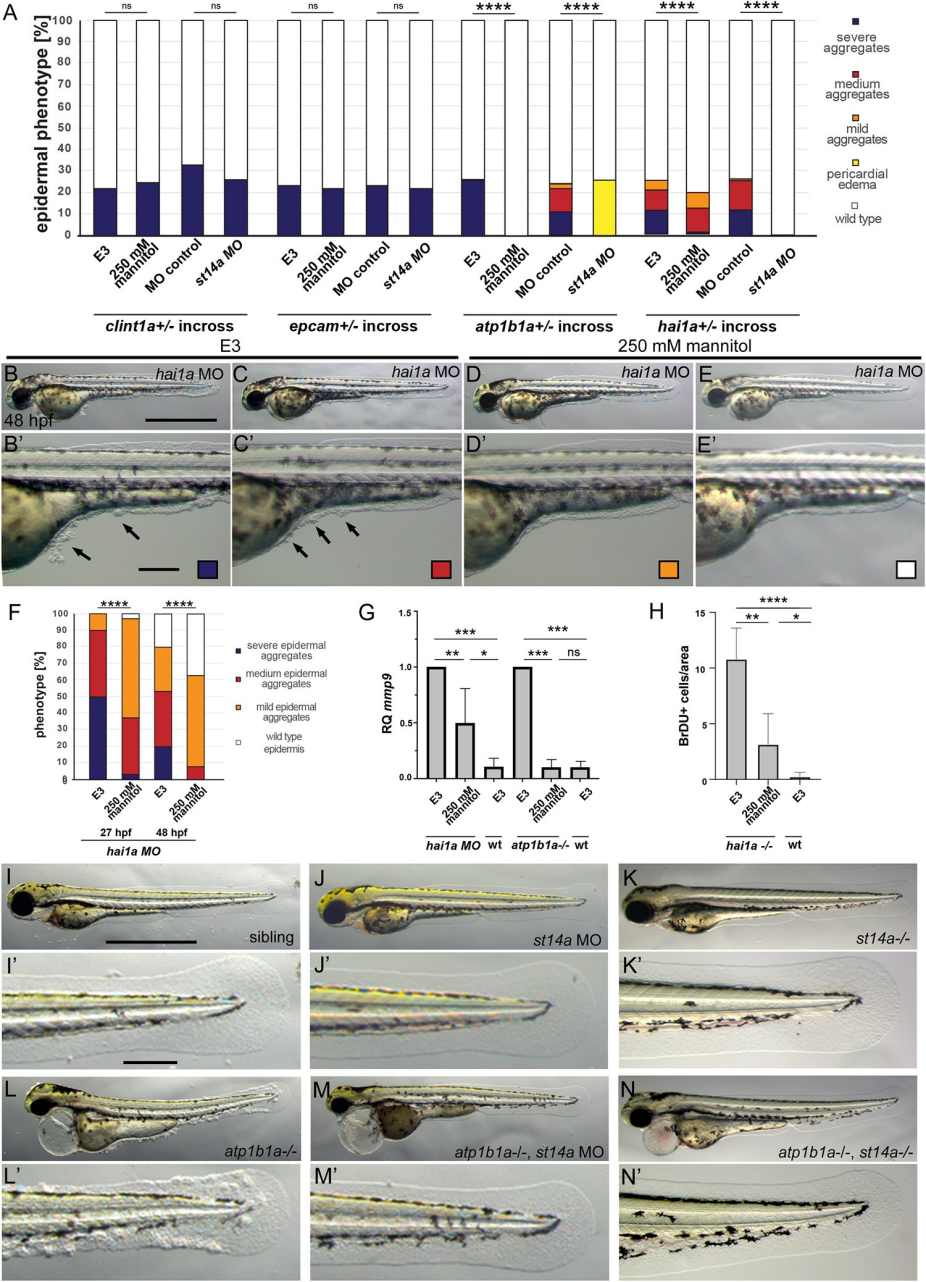
Hypotonic stress and Matriptase-1a function are required for the epidermal phenotype of *hai1a* and *atp1b1a*, but not *clint1* and *epcam* mutants. (A-H) Isotonic medium attenuates the phenotype of *hai1a-/-* mutants and rescues the phenotype of *atp1b1a-/-* mutants, but has no effect of *clint1-/-* or *epcam-/-* mutants. (A) Quantification of epidermal phenotypes of 48 hpf embryos obtained from parents heterozygous for the mutations *clint1*^*hi1520*^ [22], *epcam*^*hi2151*^ [42], *atp1b1a*^*m14*^ [26], or *hai1a*^*hi2217*^ [21], raised in E3, E3 + 250 mM Mannitol, or injected with control morpholino or *st14a* morpholino (n = 59–102 from N = 3 independent clutches per condition, Significances were determined via a Chi-square test, ns, not significantly different (p>0.05); ****, significantly different (p< 0.0001)). (B-E’) Brightfield images of live 48 hpf *hai1a* morphants raised in E3 (B,B’,C,C’) or E3 + 250 mM Mannitol (D,D’,E,E’) as lateral overviews of entire embroys (A-D) or magnified lateral views of the yolk tube and yolk extension regions of the same embryos (A’-D’); arrows in (B’,C’) point to epidermal aggregates on yolk sac and ventral median fin fold. (F) Quantification of phenotypic classes (n = 27–67) of *hai1a* morphant embryos raised in E3 or E3 + 250 mM Mannitol, representatives of which are shown in panels (B-E’) Significances were determined via a Chi-square test, ns, not significantly different (p>0.05); ****, significantly different (p< 0.0001). (G) RT-qPCR showing relative quantities of *mmp9* transcript of 48 hpf *hai1a* morphant or 56 hpf *atp1b1a* mutant embryos raised in E3 or E3 + 250 mM Mannitol, compared to their respective siblings. cDNA was obtained from pools of 15 embryos each, N = 3 for *hai1a*, N = 3 for *atp1b1a*. H. Quantification of BrdU-labeled nuclei in defined, equally-sized areas of the fin fold of wild types and *hai1a-/-* mutants at 48 hpf, raised in E3 or E3 + 250 mM Mannitol, n = 4–6. Significances in G and H were determined via a one-way ANOVA and Tukey’s post hoc test; ns, not significantly different (p>0.05); *,**,***,****, significantly different (p<0.05, 0.01, 0.001, 0.0001, respectively). (A,I–N’) Loss of *st14a* function rescues epidermal aggregate formation in *atp1b1a-/-* mutants. Brightfield images of representative live 72 hpf embryos, either as lateral overviews of the entire embryos (I-N), or as magnified lateral views of the tail of the same embryos (I’-N’): wild-type sibling (I,I’), wild-type sibling injected with *st14a* MO (J,J’), *st14a-/-* mutant (K,K’), *atp1b1a* -/- mutant with epidermal aggregates (L,L’), *atp1b1a* -/- mutant injected with *st14a* MO (M,M’), and *atp1b1a-/-; st14a-/-* double mutant (N,N’), both with wild-type-appearing epidermis. For quantifications, see (A) and S1I Fig. Scale bars: 500 μm (B,I), 100 μm (B’,I’).

Previously, we have shown that pre-neoplastic aggregate formation in the epidermis of zebrafish embryos lacking the Na^+^K^+^-ATPase beta subunit ATP1b1a is completely abrogated upon incubation of these zebrafish mutants in isotonic, rather than their natural hypotonic medium, pointing to a formerly largely overlooked potential oncogenic effect of hypotonic stress [26]. In this light, we wondered whether hypotonicity has a similar impact on epidermal aggregate formation caused by loss-of-function mutations in three other zebrafish genes, *hai1a*, encoding the cognate inhibitor of the type II transmembrane serine protease Matriptase-1/ St14a [21], *clint1a*, encoding a clathrin-interacting protein involved in endocytosis [22], and *epcam*, encoding an epithelial cell adhesion molecule [42]. For this purpose, embryos from incrosses of parents heterozygous for the respective mutation were raised in E3 medium, which, with an osmolality of 12 mOsm, is highly hypotonic to the zebrafish embryo, or in E3 supplemented with 250 mM Mannitol to increase the osmolality to isotonic conditions (270 mOsm) [26]. Roughly 25% of embryos obtained from *clint1a* and *epcam* heterozygote incrosses developed indistinguishable epidermal phenotypes in both hypotonic and isotonic medium (Fig 1A). In contrast, *hai1a* mutants or morphants (wild-type embryos injected with a *hai1a* antisense morpholino oligonucleotide / MO) raised in isotonic medium displayed a reduction in the severity of the epidermal aggregate phenotype compared to embryos raised in hypotonic medium. However–as opposed to *atp1b1a* mutants (Fig 1A)–the rescue was not complete (Fig 1A and 1F). Similarly, the expression of *mmp9* (Fig 1G), indicative of partial EMT of basal keratinocytes, as well as epidermal proliferation rates (Fig 1H), were attenuated in *hai1a* mutants or morphants raised in isotonic medium compared to their siblings in hypotonic medium, but–in contrast to *atp1b1a* mutants—without reaching wild-type levels (Fig 1G, [26]). Together, this indicates that hypotonic stress specifically enhances epidermal pre-neoplastic transformations caused by the loss of ATP1b1a or Hai1a, yet does not influence transformations caused by the loss of Clint1a or EpCAM.

But what do ATP1b1a and Hai1a have in common that makes them susceptible to environmental tonicity? In line with the role of Hai1 as the cognate inhibitor of Matriptase-1, the epidermal defects in *hai1a* mutants are completely restored to wild-type conditions by inactivating Matriptase-1 with *st14a* MOs (Fig 1A) [21]. As former reports have demonstrated a positive effect of low ionic strength on the proteolytic activity of Matriptase-1 *in vitro* [10,11,12], we wondered whether the partial rescue of *hai1a* mutants by isotonicity might be due to an isotonicity-induced reduction in Matriptase activity. Conversely, the epidermal defects of *atp1b1a* mutants might be due to a hypotonicity-induced increase in Matriptase activity, whereas the defects of *clint1a* and *epcam* mutants are Matriptase-independent. To look into the latter, offspring from incrosses of *clint1a*, *epcam*, *atp1b1a* or *hai1a* heterozygotes were injected with *st14a* MO to inactivate endogenous Matriptase-1 and raised in hypotonic E3 medium. Indeed, *st14a* MO-injected *atp1b1a* mutants displayed a complete rescue of the epidermal aggregate phenotype (Fig 1A, 1I, 1J, 1L and 1M; but persistent pericardial edema, see below), similar to the response of *hai1a* mutants (Fig 1A) [21], whereas *clint1a* and *epcam* mutants did not respond (Fig 1A). To validate these data, we also generated a genetic *st14a* null mutant (*st14a*^*fr56*^), using CRISPR/Cas9 technology (S1A–S1C Fig), to genetically delete Matriptase-1 function in *atp1b1a* mutants. Strikingly, *atp1b1a-/-; st14a-/-* double mutants (Figs 1K and 1N and S1D and S1E), as well as embryos homozygous for *atp1b1a* but heterozygous for the mutant *st14a*^*fr56*^ allele (S1H, S1I and S1J Fig), did not develop any epidermal aggregates, implying a haploinsufficiency of *st14a* in this context. Similarly, only 38% of *st14a*+/- embryos injected with *hai1a* MO developed aggregates, compared to 0% of injected *st14a*-/- embryos (S1F, S1G, S1H and S1K Fig) and 100% of injected embryos homozygous for the wild-type *st14a* allele (S1H and S1K Fig).

Together, this indicates that Matriptase-1 acts, in a dose-dependent manner, downstream of both Hai1a and ATP1b1a to mediate the pre-neoplastic effects of their losses in the embryonic epidermis.

### Matriptase is required for the activation of the oncogenic pathway in basal keratinocytes of *atp1b1a* mutants

**Fig 2:**
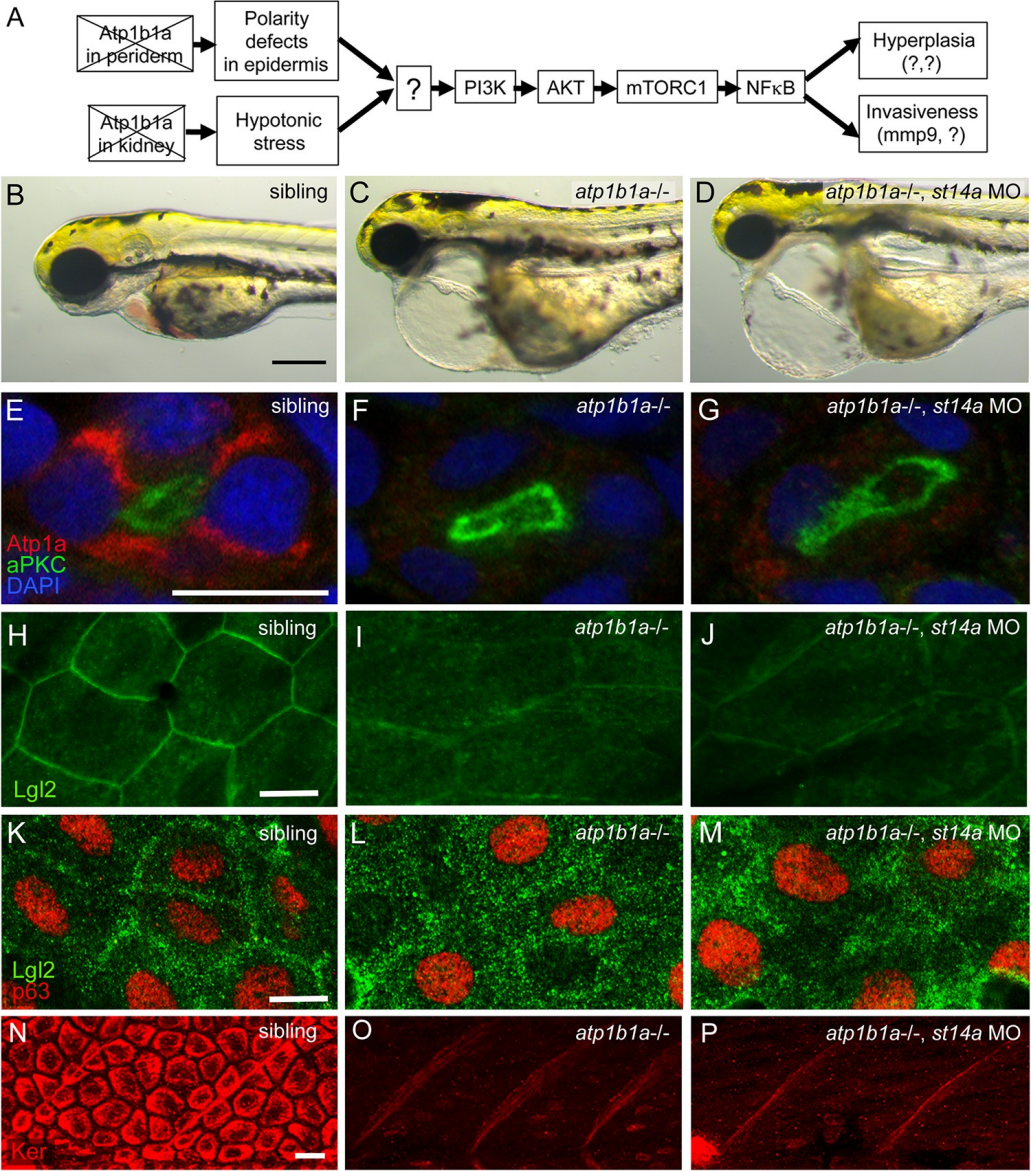
Matriptase-1 functions downstream of epidermal polarity defects and hypotonic stress induced by loss of ATP1b1a. (A) Schematic of the previously [26] identified tumorigenic pathway activated in basal keratinocytes downstream of ATP1b1a loss. (B-D) Live brightfield images of 72 hpf embryos displaying pericardial edema both in *atp1b1a* mutants (D) and *atp1b1a* mutants injected with *st14a* MO (E) but epidermal aggregates only in *atp1b1a* mutants. (E-G) Immunofluorescence for Atp1a and aPKC on cross-sections of 54 hpf *atp1b1a* sibling (E), *atp1b1a* mutant (F), and *atp1b1a* mutant injected with *st14a* MO (G) showing absence of Na^+^,K^+^-ATPase on the basolateral sides of epithelial cells of the pronephric ducts (apically labelled by aPKC) in both *atp1b1a-/-* and *atp1b1a-/-*, *st14a* MO (N = 3, n = 23–36). (H-M) Immunofluorescence for Lgl2 (green) and p63 (red) on whole mounts of 54 hpf *atp1b1a* sibling (H, peridermal layer, K, basal layer), *atp1b1a* mutant (I, peridermal layer, L, basal layer), and *atp1b1a* mutant injected with *st14a* MO (J, peridermal layer, M, basal layer), lateral views on trunk regions (N = 3, n = 35–41). (N-P) Immunofluorescence for panKeratin (Ker, red), on whole mounts of 84 hpf *atp1b1a* sibling (N), *atp1b1a* mutant (O), and *atp1b1a* mutant injected with *st14a* MO (P), lateral views on trunk regions (N = 3, n = 24–28). Scale bars: 100 μm (B), 10 μm (E,H,K,N).

While the functional correlation between Hai1 and Matriptase-1 is well understood [1,3], virtually nothing is known about the connection between ATP1b1a and Matriptase-1. We first wondered where exactly Matriptase-1 is located within the formerly reported pathway downstream of ATP1b1a [26] (Fig 2A). Strikingly, *atp1b1a*, *st14a* double mutant embryos still exhibit pericardial edema formation (Fig 1L–1N, S1I, S1J and 2B–2D) and die around 120 hours post fertilization (hpf), demonstrating that the concomitant loss of Matriptase-1 activity does not rescue hypotonic stress, nor defects in heart and kidney function of *atp1b1a* mutants. Indeed, as in *atp1b1a* single mutants, the α-subunit of the Na/K-ATPase is still absent in the pronephric duct of *atp1b1a*, *st14a* double mutants (Fig 2E–2G; n = 23–36). Similarly, double mutants still display the epithelial polarity defects formerly described for *atp1b1a* single mutants [26], indicated by reduced localization of Lgl2 to the plasma membrane in peridermal (Fig 2H–2J) as well as basal cells (Fig 2K–2M; n = 35–41) at 54 hpf, and the absence of Keratin in basal cells at 84 hpf (Fig 2N–2P; n = 24–28). Together this suggests that Matriptase-1 acts downstream of the hypotonic stress caused by loss of ATP1b1a in the kidney, and downstream of the epithelial polarity defects caused by loss of ATP1b1a in the epidermis (Fig 2A).

**Fig 3:**
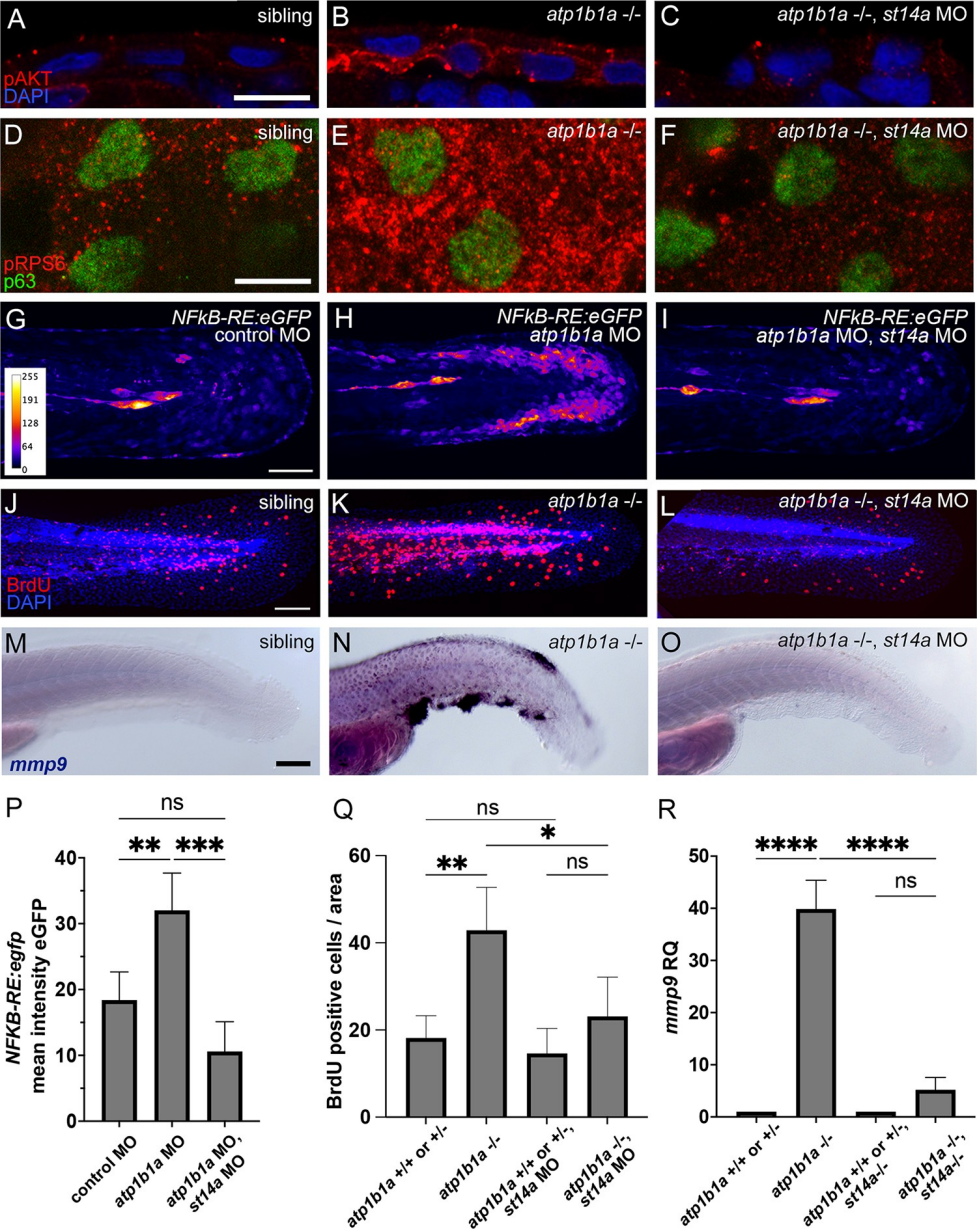
Matriptase-1 functions upstream of PI3K-AKT-mTORC1-NFkB to induce hyperproliferation and partial EMT. (A-C) Immunofluorescence on cross-sections of 54 hpf *atp1b1a* sibling (A), *atp1b1a* mutant (B), and *atp1b1a* mutant injected with *st14a* MO (C) showing pAKT (red) in basal cells in *atp1b1a-/-* but not in *atp1b1a-/-*, *st14a MO* (N = 3, n = 41–46). (D-F) Immunofluorescence on whole mounts, lateral views on trunk region for pRPS6 (red) and tp63 (green) in 54 hpf *atp1b1a* sibling (D), *atp1b1a* mutant (E), and *atp1b1a* mutant injected with *st14a* MO (F) (N = 3, n = 27–32). (G-I) Confocal images of the tail region of live 54 hpf embryos transgenic for NFkB-RE:eGFP injected with control MO (G), injected with *atp1b1a* MO (H) or co-injected with *atp1b1a* and *st14a* MO (I). Intensity of the GFP signal is color-coded. (J-L) Immunofluorescence of BrdU incorporation (red) of 56 hpf wild-type sibling (J), *atp1b1a* mutant (K), and *atp1b1a* mutant injected with *st14a* MO (L). (M-O) Whole mount *in situ* hybridization of *mmp9* in 58 hpf wild-type sibling (M), *atp1b1a* mutant (N), or *atp1b1a* mutant injected with *st14a* MO (O) (N = 2, n = 18–24). (P) Quantification of GFP signal obtained from the fin fold region of embryos as shown in (G-I) (n = 3–5). (Q) Quantification of BrdU-positive cells in the fin fold area (n = 3–5). (R) RT-qPCR showing relative quantities of *mmp9* transcript of 58 hpf *atp1b1a-/-* mutants in comparison to their *atp1b1a+/+ and atp1b1a+/-* siblings, either containing (bars 1+2) or lacking (*st14a-/-*; bars 3+4) functional Matriptase-1 (cDNA obtained from 15 pooled embryos each, N = 3). Significances in (P-R) were determined via a one-way ANOVA and Tukey’s post hoc test; ns, not significantly different (p>0.05); *,**,***,**** significantly different with p<0.05, p<0.01, p<0.001, p<0.0001, respectively. Scale bars: 10 μm (A,D,G,J,M).

Whereas these two phenotypic traits of *atp1b1a* mutants persist upon concomitant loss of St14a, components of the ATP1b1a-dependent oncogenic pathway downstream of the convergence point of the epithelial polarity and osmoregulatory branches are affected [26] (Fig 2A). *atp1b1a* single mutants display elevated levels of pAKT in basal cells as a consequence of upregulated PI3K signaling, which in turn leads to increased mTORC1 and NFkB activity [26] (Fig 2A). Concomitant loss of Matriptase-1 activity, however, reduces AKT phosphorylation (Fig 3A–3C; n = 41–46), mTORC1 activity (reflected by pRPS6 immunofluorescence as a readout for mTORC1 signaling; Fig 3D–3F; n = 27–32), as well as transcriptional NFkB activity (reflected by the intensity of eGFP in a NFkB-RE:eGFP transgenic line; Fig 3G–3I, 3P), back to wild-type levels. Furthermore, loss of Matriptase-1 function in *atp1b1a* mutants blocks downstream effects of NFkB, such as the hyper-proliferation in the epidermis (Fig 3J–3L, 3Q) and the strongly elevated expression of *mmp9*, indicative of partial EMT (Fig 3M–3O, 3R), both of which contribute to the pre-neoplastic transformation of basal keratinocytes caused by the loss of ATP1b1a [26].

This indicates that Matriptase acts upstream of the aberrantly activated PI3K-AKT-mTORC1-NFkB pathway in basal keratinocytes of *atp1b1a* mutants. As epithelial polarity and osmoregulatory defects still persist (Fig 2), Matriptase-1 represents the thus far unknown mediator downstream of the loss of ATP1b1a, where the epithelial polarity and osmoregulation branches converge to activate the oncogenic PI3K-AKT-mTORC1-NFkB pathway [26] (Fig 2A).

### Par2b is partially required for epidermal carcinogenesis in *atp1b1a* mutants

In the context of the loss of its cognate inhibitor Hai1, Matriptase-1 has been shown to act via the G-protein coupled receptor Par2. Upon proteolytic cleavage of its ectodomain by Matriptase-1, Par2 can activate different intracellular signal transduction pathways [43,44,45,46]. Accordingly, in zebrafish *hai1a* mutants, simultaneous knockdown or knockout of Par2b can almost completely restore the wild-type phenotype [20,23,25]. In *atp1b1a* mutants, concomitant loss of Par2b function via *par2b* MOs or a genetic *par2b*^*fr57*^ null mutation generated via CRISPR/Cas9 technology (S2J–S2L Fig) also attenuates epidermal aggregate formation and *mmp9* expression in basal keratinocytes in a dose-dependent manner (S2A–S2I Fig). However, in contrast to *hai1a* mutants treated in parallel (S2M–S2Q Fig), this rescue is incomplete, and wild-type conditions are not fully restored. We conclude that also in *atp1b1a* mutants, the oncogenic effect of Matriptase-1 is–at least in part–mediated by Par2b. However, in contrast to *hai1a* mutants and the activation of the EGFR-PLD pathway [20], additional Matriptase-1 targets seem to be involved to fully activate the ATP1b1a-specific oncogenic pathway via PI3K and AKT.

### In cultured human cells, hypotonic medium and loss of the epithelial cell polarity regulator Scribble increase Matriptase-1 activity

**Fig 4:**
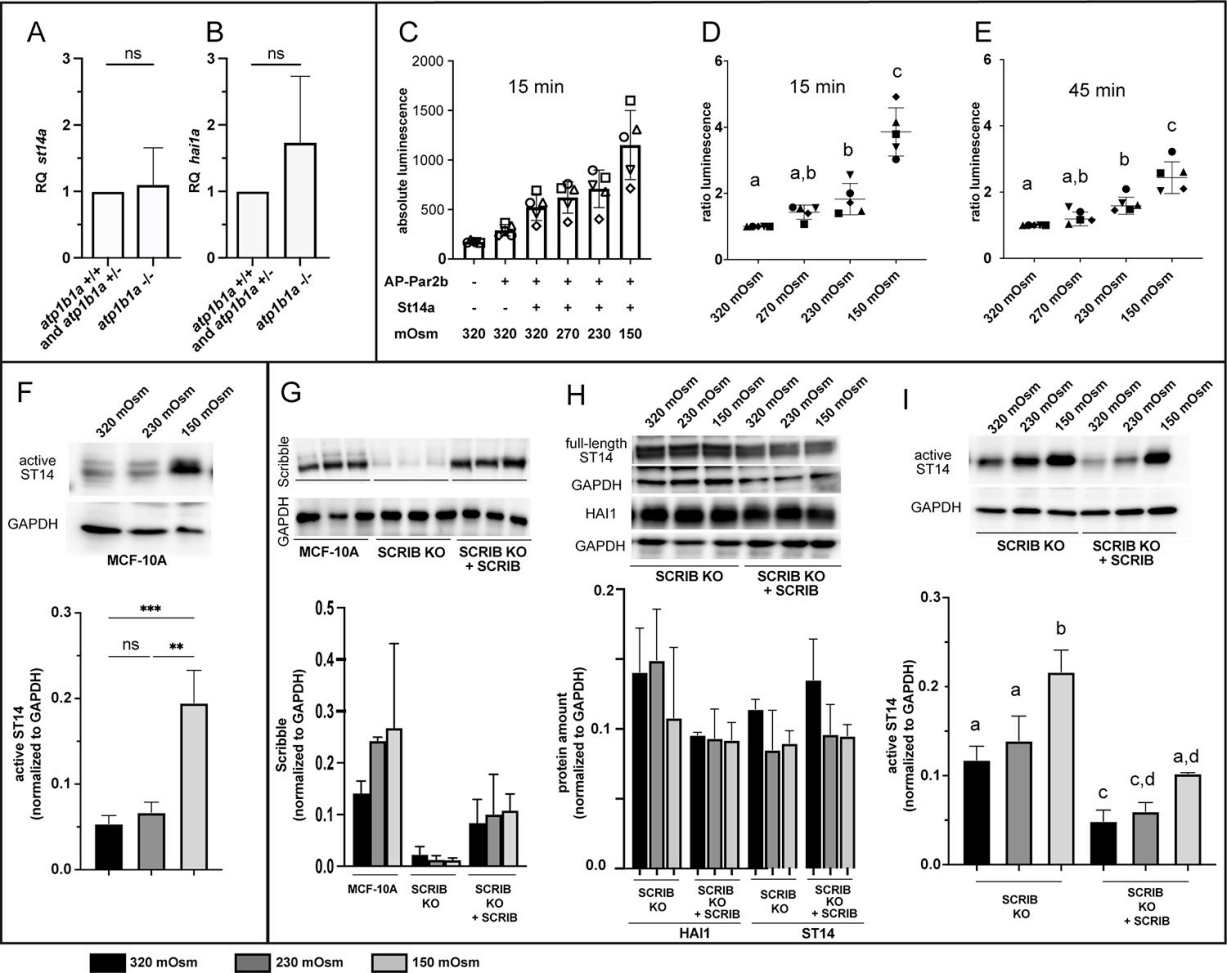
Matriptase activity is increased by hypotonicity and polarity defects. (A-B) RT-qPCR showing no significant change of *st14a* and *hai1a* transcript levels in *atp1b1a* mutants compared to their siblings (n = 3, cDNA obtained from pools of 15 embryos each at 56 hpf, significances were determined via Student’s t-test; ns, non-significant difference; p>0.05). (C-E) Reporter assay for Matriptase activity towards Par2 cleavage showing that zebrafish Matriptase1a cleaves AP-Par2b more efficiently at lower osmolalities. HEK293 cells were transfected with empty pcDNA3, pcDNA3+AP-Par2b, and pcDNA3+St14a. After 24 hrs in regular / isotonic medium (tonicity of 320 mOsm), cells were exposed to media of 320 mOsm, 270 mOsm, 230 mOsm, and 150 mOsm for 15 and 45 minutes, respectively. (C) At 15 min, absolute luminescence values of AP released into the supernatant progressively increase with increasing hypotonicity / lower tonicity. (D,E) Ratios of luminescence between isotonic and hypotonic media indicate an up to 3.79-fold increase (in 150 mOsm medium) after 15 min (D) and a 2.51-fold increase after 45 min (E). Ratios were determined from values as shown in (C), deducting baseline luminescence (bar 1 in C) from the luminenscences of co-transfected samples obtained at different osmolarities (bars 3–6 in C), normalized against the value at 320 mOsm (isotonic); n = 5, significances were determined via a one-way ANOVA and Tukey’s post hoc test; columns with same superscript letter (a,b,c) are not significantly different (p>0.05). (F) Immunoblot analysis for processed / activated Matriptase-1 (ST14) showing that culturing of MCF-10A cells for 24 hours in hypotonic medium (150 mOsm) leads to a 3.78-fold increase in active endogenous Matriptase-1 compared to cells cultured in isotonic medium (320 Osm). Bar diagram displays mean value of proteins normalized to loading control GAPDH (n = 3); ns, not significantly different (p>0.05); **, ***, significantly different with p<0.01, p<0.001, respectively. (G-H) Scribble knockout (SCRIB KO) in human MCF-10A cells does not affect protein levels of full length ST14 or HAI1. Representative western blots show unaltered amounts of endogenous full-length ST14 (G) and HAI1 (H) proteins in SCRIB-KO cells compared to knockout cells with re-introduced Scribble (SCRIB KO + SCRIB), cultured in media of 320 mOsm, 230 mOsm, or 150 mOsm for 1 hr. Bar diagrams display mean value of proteins normalized to loading control GAPDH (n = 2); all differences are not statistically significant (p>0.05). (I) Loss of Scribble increases active ST14 in media of different osmolalities. Representative western blots showing active ST14 and GAPDH of SCRIB-KO and SCRIB-KO + SCRIB cells cultured in media of 320 mOsm, 230 mOsm, and 150 mOsm for 24 hrs, with highest numbers in cells lacking the epithelial polarity protein Scribble protein and exposed to hypotonic stress. Bar diagram displays mean value of proteins normalized to loading control GAPDH (n = 3). Significances were determined via a one-way ANOVA and Tukey’s post hoc test; columns with same superscript letter (a,b,c,d) are not significantly different (p>0.05).

We next aimed to elucidate the molecular and cellular mechanisms by which loss of ATP1b1a leads to aberrantly increased Matriptase-1 activity. In zebrafish *hai1a* mutants and multiple human malignancies [1,13], this hyper-activity most likely results from an imbalanced ratio between Matriptase-1 and its cognate inhibitor Hai1. In zebrafish *atp1b1a* mutants, however, *st14a* and *hai1a* transcript levels are not significantly changed compared to their siblings (Fig 4A and 4B), pointing to other mechanisms. If Matriptase-1 is indeed positioned downstream of the hypotonic stress induced by the loss of ATP1b1a, as proposed above (Fig 2A), its activity should—even in systems with wild-type ATP1b1 function—be influenced by the ionic strength of the environment and by epithelial cell polarity regulators. To analyze this and to look into the evolutionary conservation of this mechanism, we turned to mammalian cell culture systems.

To test whether hypotonic stress increases the activity of zebrafish Matriptase-1 towards cleavage of its likely *in vivo* substrate Par2b, we applied a formerly established HEK293 cell culture assay. Hereby, the activity of Matriptase-1 is measured by the cleavage of the extracellular domain of its substrate Par2b fused to Alkaline Phosphatase (AP), quantifying released AP in the supernatant via an enzymatic reaction [25,44,47]. Previous work via this assay has demonstrated that, similar to mammalian counterparts, zebrafish Matriptase-1a cleaves Par2b mainly at the canonical cleavage site [25]. Here, cells co-transfected with plasmids encoding zebrafish Matriptase-1a and AP-Par2b were grown in isotonic medium for 24 hours and then exposed to media of different osmolalities (320 mOsm as the standard cell culture condition control, 270 mOsm corresponding to the isotonic medium zebrafish embryos were exposed to (Fig 1), and 230 mOsm and 150 mOsm as hypotonic conditions) for 15 and 45 minutes. Compared to isotonic 320 mOsm medium, a reduction of the medium osmolality to 230 mOsm resulted in a moderate increase of AP activity, and a reduction to 150 mOsm in a strong increase of AP in the supernatant already after 15 minutes, which persisted until the 45 minutes end point (Figs 4C–4E and S3). Of note, when as a control, Par2b was transfected alone, the hypotonicity-induced increase of AP release (possibly mediated by endogenous Matriptases of the HEK293 cells) was much weaker, indicating that approximately 80% of the hypotonicity-induced increase of AP release in the co-transfected samples is indeed due to an hypotonicity-induced increase in the proteolytic activity of the co-transfected zebrafish Matriptase-1a (S3 Fig).

Consistent and even more direct data pointing to a positive effect of low ionic strength on the activity of endogenous Matriptase-1 were also observed in the breast epithelial line MCF-10A, which in contrast to breast cancers [13,48,49,50] normally only contains low levels of active Matriptase-1 [9]. Active Matriptase resulting from autocatalytic cleavage of the full-length zymogen can be directly detected by immunoblot as a 26 kDa product [51]. When culturing MCF-10A cells for 24 hours in hypotonic medium, we found the levels of cleaved / active endogenous Matriptase-1 to be mildly increased under moderate (230 mOsm) and more strongly increased under strong (150 mOsm) hypotonic conditions (Fig 4F).

Together, these results imply hypotonic stress as a factor enhancing the proteolytic activity of both zebrafish and human Matriptase-1, consistent with former data obtained for the activity of mammalian Matriptase-1 towards other substrates [10,11,12], and suggesting that in zebrafish *atp1b1a* mutant keratinocytes *in vivo*, Matriptase-1a activity may also be increased due to the low ionic strength conditions in the interstitial fluid of the epidermis.

Furthermore, to investigate the impact of epithelial cell polarity on human Matriptase-1 activity in this MCF-10A system, we knocked out the epithelial polarity regulator Scribble (SCRIB). Scribble is part of the Scrib/Dlg/Lgl module regulating epithelial cell polarity and has also been implicated with tumor suppression [34,37]. SCRIB knockout (KO) cells lack Scribble protein (Fig 4G), however, they do not show significant differences in the levels of full-length Matriptase-1 or HAI-1 compared to SCRIB-KO cells in which Scribble was re-introduced via transfection with the corresponding expression plasmid (SCRIB KO + SCRIB; Fig 4H). Intriguingly, after 24 hours of incubation in different osmolalities, the positive effect of hypotonic stress on Matriptase-1 activity was significantly stronger in SCRIB KO cells compared to SCRIB KO + SCRIB cells (Fig 4I). These data implicate that also in mammalian cells, disrupted epithelial cell polarity has Matriptase-activating capacity, which synergizes with the effect of hypotonic stress, similar to their proposed cooperation in zebrafish *atp1b1a* mutants.

### Matriptase-1a functions in the periderm to activate oncogenic signaling in the underlying basal layer

**Fig 5:**
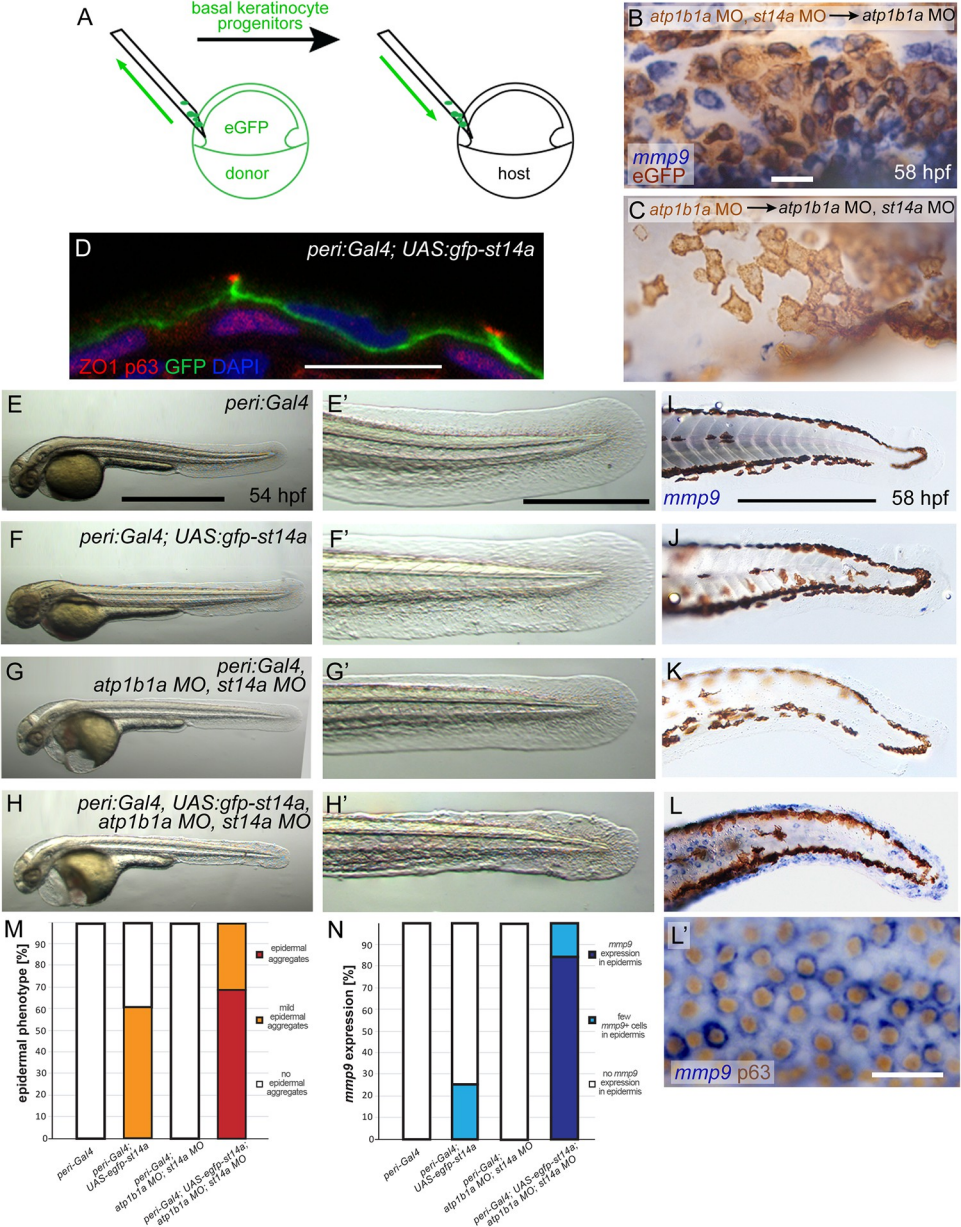
St14a functions in the periderm to activate signaling in the underlying basal layer. (A-C) Loss of *st14a* in basal cells is neither necessary nor sufficient to normalize *mmp9* expression in basal cells of *atp1b1a* morphants. (A) Schematic of experimental set up in which ventral ectodermal cells either from *atp1b1a*, *st14a* double morphant or *atp1b1a* morphant donors were homotopically transplanted at 6 hpf into the same region of *atp1b1a* or *atp1b1a*, *st14a* double morphant hosts, respectively (*atp1b1a* MO, *st14a* MO>*atp1b1a* MO or *atp1b1a* MO>*atp1b1a* MO, *st14a* MO). Donor cells express *Tg(Ola*.*Actb*:*Hsa*.*hras-egfp)-*encoded membrane-tagged eGFP. Whole mount *in situ* hybridization for *mmp9* (blue) and immunohistochemistry for eGFP (brown) of chimeric embryos at 58 hpf, showing that in *atp1b1a* MO, *st14a* MO>*atp1b1a* MO experiments, *atp1b1a* MO, *st14a* MO basal cells express *mmp9* (B), and are thus not rescued although they lack functional Matriptase-1; whereas in *atp1b1a* MO>*atp1b1a* MO, *st14a*MO experiments, *atp1b1a* MO basal cells lack *mmp9* expression (C), and are thus rescued although they contain Matriptase-1 (n = 6–8). (D-N) Re-introduction of *st14a* into peridermal cells of *atp1b1a*, *st14a* double morphants abrogates the rescue of basal cells, reflected by re-gained epidermal aggregate formation and enhanced *mmp9* expression. (D) Immunofluorescence for eGFP-Matriptase1a (green), p63 as a nuclear marker for basal cells (red) and ZO1/Tjp1 as a marker for tight junctions in peridermal cells (red) on transverse section through the epidermis of a 48 hpf embryo transgenic for *peri*:*Gal4*^*zc1044a*^ and *UAS*:*gfp-st14a*^*fr58Tg*^, counterstained with DAPI (blue). Transgene-encoded Matriptase-1 is restricted to peridermal cells and localized at their basolateral membranes. (E-H’) Brightfield images of representative live 56 hpf embryos transgenic for *peri*:*Gal4* ^*zc1044a*^ or *peri*:*Gal4* ^*zc1044a*^*; UAS*:*eGFP-st14a* controls (E,F) and injected with *atp1b1a* and *st14a* morpholinos (*atp1b1a* MO, *st14a* MO) (G,H); lateral views of entire embryos (E-H), and magnified views of tail region of same embryos (E’-H’). In contrast to *atp1b1a* MO, *st14a* MO embryos without transgenic re-introduction of *st14a* (G,G’), the *atp1b1a* MO, *st14a* MO embryos transgenic for *peri*:*Gal4*^*zc1044a*^*; UAS*:*eGFP-st14a* displays epidermal aggregates (H,H’), comparable to global *atp1b1a* single mutants (compare with Fig 1L’). (I-L’) Representative whole mount *mmp9 in situ* hybridizations, revealing re-gained strong *mmp9* expression in basal cells of 72 hpf *atp1b1a* MO, *st14a* MO embryo transgenic for *peri*:*Gal4*^*zc1044a*^*; UAS*:*eGFP-st14a* (L,L’, the latter counterstained for the basal cell marker p63), but not in the *atp1b1a* MO, *st14a* MO embryos lacking transgene-driven peridermal *st14a* re-expression (K). (M) Quantification of epidermal phenotype of 56 hpf embryos with respective genotypes, as shown in (E-H) (n = 20–46). (N) Quantification of *mmp9 in situ* signal of 72 hpf embryos with respective genotypes, as shown in (I-L) (n = 12–38). Scale bars: 20 μm (B,L’), 10 μm (D), 500 μm (E), 100 μm (E‘,I).

To understand by which means epithelial polarity affects Matriptase function, we returned to the zebrafish embryos. Previous studies have shown that in the epidermis of wild-type embryos, the Matriptase-1 encoding *st14a* gene is expressed in both layers of the epidermis [21,25]. In contrast, *atp1b1a* is only expressed in the outer peridermal layer, in which it, however, is required to assure proper epithelial polarity in keratinocytes of both layers [26]. In this light, we first sought to identify the epidermal layer in which Matriptase-1 activity needs to be hyper-activated to mediate the oncogenic effects caused by the loss of ATP1b1a function. For this purpose, in a first set of experiments, we performed cell autonomy studies with mosaic embryos. Two types of chimeric embryos were generated via transplantation of ventral ectodermal cells, the progenitors of basal cells (Fig 5A), yielding clones of basal keratinocytes lacking both ATP1b1a and Matriptase-1 positioned underneath an ATP1b1a-deficient periderm containing Matriptase-1a (Fig 5B), and vice versa (Fig 5C). In both cases, transplanted basal cells at 58 hpf display *mmp9* expression levels according to the genetic constitution of the host periderm. Thus, loss of Matriptase-1 in the host / the periderm (*atp1b1a* MO, *st14a* MO) rescues clones of underlying basal cells (*atpb1a* MO) to wild-type condition, even though they do contain Matriptase-1 (no *mmp9* expression in basal cells in brown to label eGFP protein; Fig 5C), whereas loss of Matriptase-1 in clones of basal cells themselves (*atp1b1a* MO, *st14a* MO) leaves them pre-neoplastic (strong *mmp9* expression in basal cells labelled in brown; Fig 5B), as long as the host / the overlying periderm contains (unrestrained) Matriptase-1 activity (*atpb1a1* MO).

Together, these data indicate that *st14a*, although it affects basal keratinocytes, does not act in basal keratinocytes themselves, but in other cells, most likely in the overlying periderm. To directly prove the latter, we employed a transgenic approach (*peri*:*Gal4* [52];*UAS*:*gfp-st14a*) to specifically re-introduce Matriptase-1a fused with GFP into peridermal cells of *atp1b1a*,*st14a* double-deficient embryos. Immunofluorescence analysis confirmed that GFP-Matriptase1a was solely present in the periderm, where it is localized in lateral and basal plasma membrane domains (Fig 5D). Driving peridermal expression of *gfp-st14a* in otherwise wild-type embryos has a mild effect on the epidermis, with minor aggregate formation (Fig 5E, 5F and 5M) and undetectable *mmp9* expression (Fig 5I, 5J and 5N). Expression of *gfp-st14a* in *atp1b1a*,*st14a* double morphants, however, which *per se* also have an epidermis of wild-type appearance (Fig 5G, 5K, 5M and 5N), leads to strong aggregate formation and *mmp9* expression in basal keratinocytes (Fig 5H, 5L, 5M and 5N), comparable to the epidermal defects in regular *atp1b1a* mutants (compare with Figs 1M and 3N).

Together, these data suggest that loss of ATP1b1a function in peridermal cells leads to increased signaling of Matriptase-1 from peridermal cells to underlying basal keratinocytes, thereby activating the oncogenic pathway and *mmp9* expression in basal cells. This would be in line with cell non-autonomous functions of Matriptase described before in other contexts, for example in oral squamous cell carcinomas (OSCC), in which deregulated Matriptase from the cancer cells was suggested to activate Par2 on adjacent tumor-associated fibroblasts [53].

### Epithelial polarity defects result in disrupted subcellular localization of Matriptase-1 in the periderm and affect Par2b in basal keratinocytes

**Fig 6:**
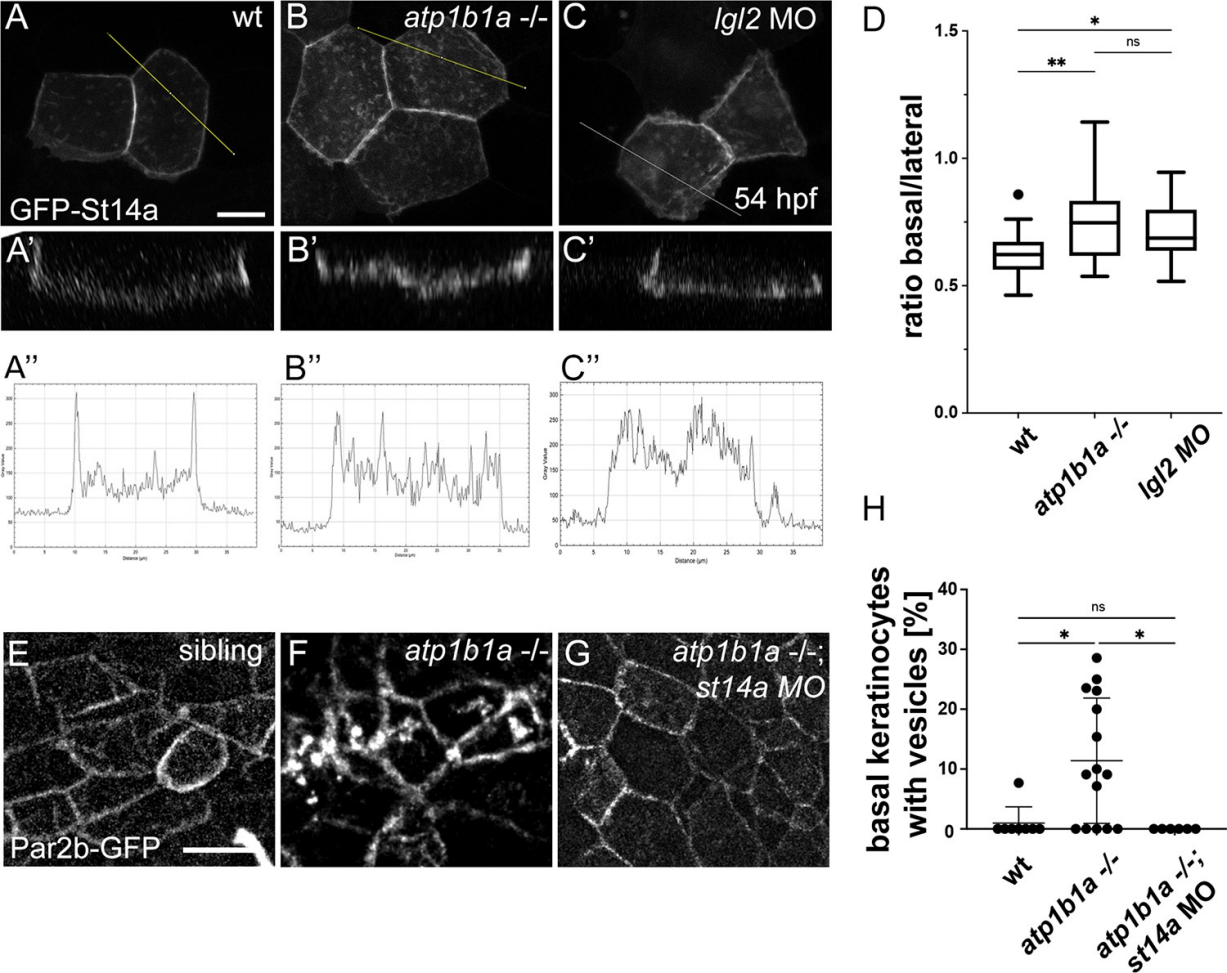
Epithelial polarity defects lead to altered distribution of Matriptase-1 within the basolateral domain of peridermal cells and of Par2b in basal cells. (A-D) *atp1b1a* mutants and *lgl2* morphants display a shift of eGFP-St14a from lateral towards basal sides of peridermal cell membranes. (A-C’) Live confocal z-stack images of eGFP-St14a in peridermal cells of 48 hpf, *krt4*:*egfp-st14a*-injected wild-type (A), *atp1b1a-/-* (B), and *lgl2* MO (C) embryos, presented as sum slices projections (A-C) and orthogonal views (A’-C’); scale bar: 10 μm. (A”-C”) Grey values obtained from the plot profile of yellow lines crossing cells in A-C. (D) Quantification of the ratio of the mean intensity of basally versus laterally localized eGPF (n = 15–30; significances were determined via a one-way ANOVA and Tukey’s post hoc test; ns, not significantly different (p>0.05); *,**, significantly different with p<0.05, p<0.01, respectively). (E-H). Basal cells of *atp1b1a* mutants display *st14a*-dependent localization of Par2b-eGFP in intracellular vesicles. Maximal intensity projections of live confocal z-stacks of basal cells in the fin fold region in wild-type siblings (E), *atp1b1a* mutants (F), and *atp1b1a* mutants injected with *st14a* MO (G), all transgenic for *Tg(ΔNp63*:*par2b-egfp)*^*fr59Tg*^; scale bar: 20 μm. All embryos display Par2b-GFP at the lateral membranes of basal keratinocytes. The wild-type sibling and *st14a*-deficient *atp1b1a* mutant display a rather homogeneous faint staining in the “interior” of cells (E,G), which might represent Par2b-GFP at the apical cell membranes, and which is even weaker in the *atp1b1a* mutant (F). On the other hand, some, but not all, basal keratinocytes of the *atp1b1a* mutant contain larger and roundish intracellular Par2b-GFP-positive structures (F), which might represent vesicles and which are absent from the wild-type and *st14a*-deficient *atp1b1a* mutant embryo (E,G). (H), Quantification of basal cells containing GFP-positive vesicle-like figures per embryo (n = 6–15 embryos; significances were determined via a one-way ANOVA and Tukey’s post hoc test; ns, not significantly different (p>0.05); *, significantly different (p<0.05)).

But how does loss of ATP1b1a in peridermal cells cause increased Matriptase-1 signaling to underlying basal keratinocytes? Could the loss of Atp1b1a as a regulator of epithelial cell polarity affect Matriptase-1 localization in peridermal cells and thereby Par2b in underlying basal keratinocytes? In mammalian cells, Matriptase-1 has been shown to be targeted to basolateral membranes [54,55], co-localizing with E-cadherin and basolateral determinants like Na^+^K^+^-ATPase. To analyze Matriptase-1 localization in the zebrafish periderm, 1-cell stage embryos were injected with a *krt4*:*egfp-st14a* –containing plasmid to express the GFP-Matriptase-1 fusion protein (Fig 5D) specifically in single peridermal cells (Fig 6A–6D). Orthogonal views and quantifications on sum of slices projections demonstrate that in wild-type embryos, the fusion protein is mainly localized at lateral and less at basal cell membranes (Fig 6A’,6A” and 6D), whereas in *atp1b1a* mutants (Fig 6B’,6B” and 6D) and in morphants lacking the epithelial cell polarity regulator Lgl2 [38] (Fig 6C’,6C” and 6D), relatively higher amounts of GFP are detected at the basal side. This indicates that polarity defects in the periderm as caused by the loss of ATP1b1a can result in an accumulation of Matriptase-1 on the basal side of peridermal cells, thereby bringing more of it into direct contact with potential substrates present on the apical surface of underlying basal keratinocytes.

According to our functional data described above (Figs S2 and 4), the Proteolysis-activated receptor Par2b could be one such substrate. To investigate whether it is indeed affected in basal keratinocytes of *atp1b1a* mutants, we generated a stable *tp63*:*par2b-gfp*^*fr59Tg*^ transgenic line expressing a Par2b-GFP fusion protein specifically in basal keratinocytes. In 54 hpf wild-type embryos, Par2b-GFP mainly localizes to the plasma membrane of keratinocytes (Fig 6E and 6H). Strikingly, in *atp1b1a* mutants, Par2b-GFP was additionally observed in internal vesicle-like structures of some, but not all (see Discussion) basal cells (Fig 6F and 6H). This subcellular distribution is similar to that reported for zfPar2b-GFP expressed in HEK293 cells once cells are treated with trypsin to activate Par2b, most likely reflecting activation-induced endocytic internalization of the receptor [56]. In *atp1b1a-/-*, *st14a* morphant zebrafish embryos, however, no such Par2b-GFP internalization was observed (Fig 6G and 6H), indicating that the effect depends on Matriptase-1. Together with the data described above identifying the periderm as the site of Matriptase-1 action (Fig 5), this suggests that aberrant activation of Matriptase-1 caused by the loss of ATP1b1a in peridermal cells does indeed lead to increased Par2b activation in underlying basal cells (and thereby their pre-neoplastic transformation).

### Epithelial cell polarity defects combined with hypotonic stress causes Matriptase-dependent pre-neoplastic epidermal transformations in zebrafish embryos with wild-type ATP1b1a

**Fig 7:**
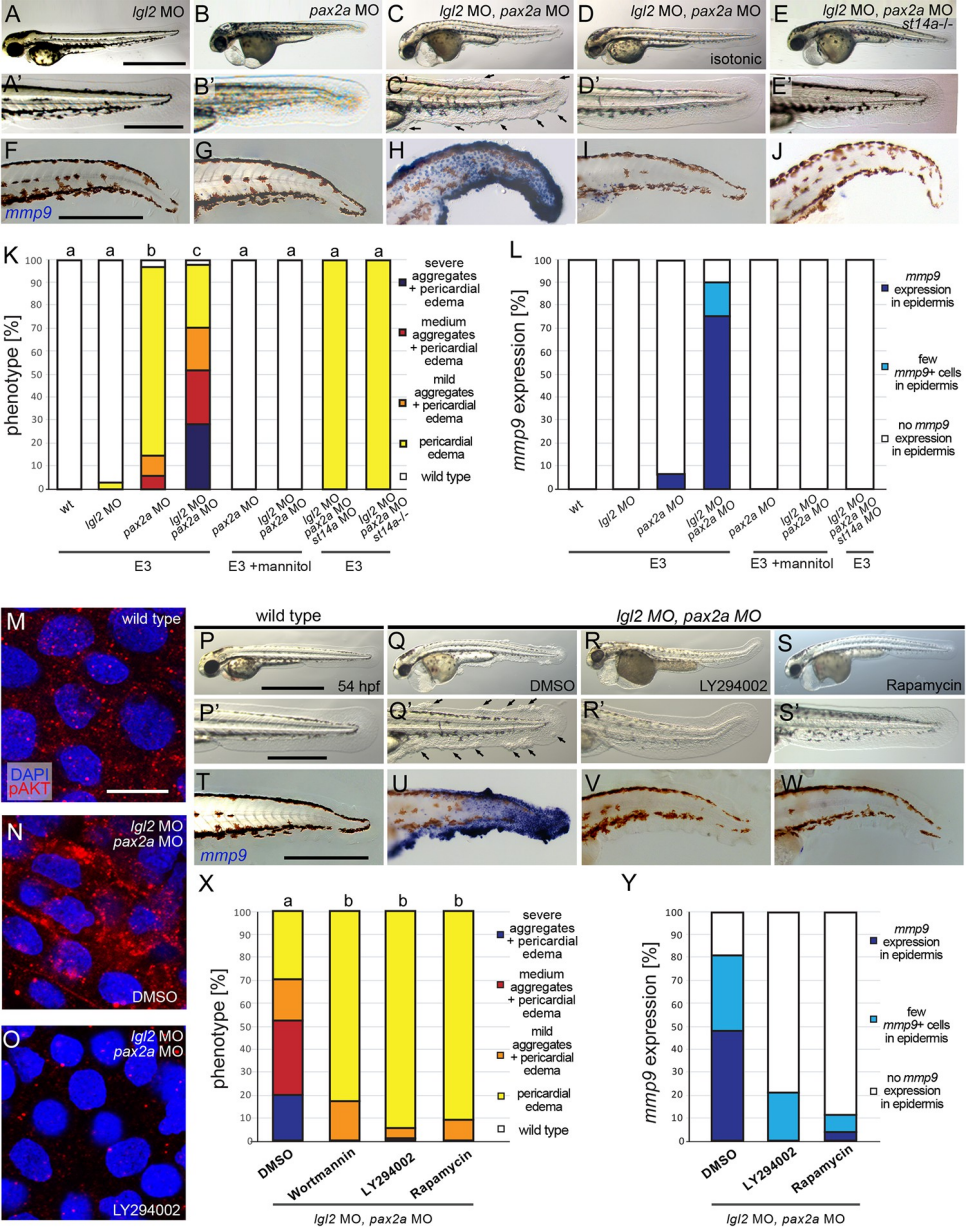
Polarity defects combined with hypotonic stress cause aberrant activation of the Matriptase-PI3K-pAKT-mTORC1 pathway and pre-neoplastic transformations of basal keratinocytes in the embryonic zebrafish epidermis. (A-E’) Brightfield images of live 54 hpf embryos morphant for *lgl2* to induce polarity defects, morphant for *pax2a* to induce hypotonic stress, or morphant for both *lgl2* and *pax2a*; overviews of entire embryos (A-E) and magnified views of tail regions (A’-E’). Whereas *lgl2* (A,A’) and *pax2a* knockdown (B,B’) alone do not result in epidermal defects, the combination of both causes epidermal aggregate formation (C,C’; indicated in C’ with arrows), which is abrogated by isotonic conditions (D,D’, raised in E3 + 250 mM Mannitol) or in *st14a* mutants (E,E’). Scale bar: 500 μm in overview, 100 μm in magnified image. (F-J) Whole mount *in situ* hybridization for *mmp9* transcripts showing *mmp9* expression only in *pax2a*, *lgl2* double morphant embryos raised in hypotonic conditions (H) but not in single morphants (F,G), or double morphants raised in isotonic conditions (I) or in a *st14a* mutant background (J). Scale bar: 100 μm. (K) Quantification of phenotypes of embryos with different genotypes / morphant conditions under hypotonic (E3) and isotonic (E3 + 250 mM Mannitol) conditions (N = 2–4, n = 22–106; significances were determined via a one-way ANOVA and Tukey’s post hoc test by comparing fraction of embryos displaying an epidermal phenotype (mild, medium, and strong) with no epidermal phenotype; columns with same superscript letter (a,b,c) are not significantly different (p>0.05).). (L) Quantification of embryos with different morphant conditions under hypotonic (E3) and isotonic (E3 + 250 mM Mannitol) conditions displaying strong *mmp9* expression, few *mmp9* positive cells, or no *mmp9* expression in the epidermis (n = 30–41). (M-O) Immunofluorescence for pAKT on whole mounts, lateral views on trunk regions, showing increased pAKT levels in basal keratinocytes in 54 hpf *lgl2*, *pax2a* double morphants (N) compared to wild-type embryo (M) or *lgl2*, *pax2a* double morphants treated with the PI3K inhibitor LY294002 (n = 12–15). (O). Scale bar: 10 μm. (P-S’) Bright field images of live 54 hpf *lgl2*, *pax2a*, double morphant embryos as overviews of entire embryos (P-S) or magnified views of tail regions (P’-S’), showing aggregate formation in the DMSO control (Q,Q; indicated in Q’ by arrows’), which is abolished by treating with 25 μM LY294002 (R, R’) or 1.1 μM Rapamycin (S,S’). Scale bar: 500 μm in overview, 100 μm in magnified image. (T-W). Whole mount *in situ* hybridization for *mmp9* transcripts showing *mmp9* upregulation in 58 hpf *pax2a*, *lgl2* double morphant in the DMSO control (U) but no *mmp9* expression in wild type (T), or in LY294002 (V) or Rapamycin (W) treated *lgl2*, *pax2a* double morphant. Scale bar: 100 μm. (X) Quantification of epidermal aggregates and pericardial edema phenotypes in embryos, representatives of which are shown in (P-S’) (n = 17–34 embryos per condition from N = 3–5 independent experiments, Significances were determined via a one-way ANOVA and Tukey’s post hoc test by comparing fraction of embryos displaying an epidermal phenotype (mild, medium, and strong) with no epidermal phenotype; columns with same superscript letter (a,b,c) are not significantly different (p>0.001).)).). (X) Quantification of embryos with widespread, scarce or no *mmp9* expression in the epidermis, representatives of which are shown in (T-W) (n = 26–29 embryos per condition from N = 3 independent experiments).

Our results described thus far have revealed that hypotonic stress and epithelial polarity defects are necessary for Matriptase-dependent pre-neoplastic epidermal transformations in the context of ATP1b1a loss of function. But are they also sufficient to do so *per se*, and can this be a more general mode of oncogenic Matriptase activation? To find out, we finally studied whether epithelial polarity defects and hypotonic stress *per se* have similar Matriptase-dependent oncogenic effects on the embryonic zebrafish epidermis even in the presence of functional ATP1b1a. To induce systemic hypotonic stress, wild-type embryos were injected with antisense MOs against *pax2a*, a transcription factor required for proper pronephros function. Zebrafish *pax2a* mutants have been described to display pronephros-specific disruption of the Na^+^/K^+^-ATPase α subunit localization. In addition, most likely as a consequence of pronephric malfunction and the resulting systemic hypotonic stress, they develop pericardial edema [57], thus defects very similar to the osmoregulatory traits of *atp1b1a* mutants. To induce epithelial polarity defects, we again targeted the cell polarity determinant Lgl2 [38,39], whose subcellular localization is disrupted in keratinocytes of *atp1b1a* mutants [26] (Fig 2H, 2I, 2K and 2L), by morpholino injection as well as using the CRISPR/Cas9 system to create F0 mutants (crispants). When injecting *pax2a* MO alone, all embryos develop pericardial edemas at 54 hpf, whereas only a small portion also develops few epidermal aggregates (Figs 7B, 7K and S4K, S4M). Similarly, knockdown or knockout of *lgl2* by itself does not result in any obvious epidermal phenotype at 54 hpf (Figs 7A, 7K and S4J, S4M), although the protein is absent (S4D–S4F Fig). In contrast, combined knockdown of *pax2a* and *lgl2* leads to a strong increase in epidermal aggregate formation (Figs 7C, 7K and S4L,S4M), and also induces *mmp9* expression in basal keratinocytes (Fig 7H and 7L), as observed in *atp1b1a* mutants (Fig 3N), whereas single loss of *pax2a* or *lgl2* does not (Fig 7F, 7G and 7L). When *pax2a* and *lgl2* double morphant embryos are raised in isotonic E3 containing 250 mM Mannitol, pericardial edema formation is strongly suppressed and epidermal aggregate formation and *mmp9* expression are completely abrogated (Fig 7D, 7I, 7K and 7L), whereas concomitant genetic or MO-induced loss of *st14a* suppresses epidermal aggregate formation and *mmp9* expression in basal keratinocytes only, but not edema formation (Fig 7E, 7J, 7K and 7L). Furthermore, basal keratinocytes of *pax2a*, *lgl2* double morphants show increased pAKT levels, indicative of PI3K signaling, while blockage of PI3K by Wortmannin or LY294002 restores wild-type pAKT levels (Fig 7M–7O) and rescues epidermal aggregate formation and *mmp9* expression (Fig 7P–7R, 7T–7V, 7X and 7Y). Similarly, inhibiting mTORC1 signaling by Rapamycin prevents epidermal aggregate formation and *mmp9* expression in basal keratinocytes (Fig 7S, 7W, 7X and 7Y). Together, this indicates that although induced by other means, loss of epithelial polarity in combination with systemic hypotonic stress activates the same Matriptase-PI3K-AKT-mTORC1 pathway resulting in epidermal hyperplasia and *mmp9* expression, as in the case of loss of ATP1b1a. This points to St14/Matriptase-1 as an oncogene that can be activated *in vivo* by multiple means and downstream of different mono- or multigenic insults to exhibit its carcinogenic properties.

## Discussion

Matriptase-1 plays a major role in epithelial development and homeostasis from zebrafish to mice and humans. It also can act as an oncogene, making its tight regulation crucial, for instance via its tumor-suppressing cognate inhibitor Hai1 [1,2]. Also in zebrafish embryos, deregulated Matriptase due to the loss of Hai1a results in preneoplastic transformations in embryonic epidermal cells. Here, we have identified a tumor-promoting role of Matriptase in a different embryonic zebrafish model: the *atp1b1a* mutant, which is characterized by similar, but not identical pre-neoplastic epidermal transformations, but interestingly does not display a reduction in *hai1a* expression, pointing to different means of oncogenic Matriptase deregulation.

### The activity of Matriptase can be increased by hypotonic stress

Matriptase is synthesized as a zymogen, which can be activated via proteolytic cleavage by other proteases, for example by Tmprss2, enhancing its effect on the growth and invasiveness of prostate cancer [58]. However, in most cases, Matriptase-1 is suggested to primarily undergo auto-activation [7]. Several non-protein factors have been shown to promote this autoactivation, including small molecules like the phospholipid sphingosine-1-phosphate [8], the small molecule suramin [59], but also reactive oxygen species (ROS) [60]. Interestingly, the cellular inorganic environment also has a crucial impact on Matriptase-1 autoactivation, which is highest in mildly acidic and low ionic strength conditions [10,11,12]. Here, we present evidence that the pericellular tonicity has a substantial effect on Matriptase activation and activity, both in zebrafish embryos *in vivo* as well as in cultured human embryonic kidney (HEK293) and breast epithelial (MCF-10A) cells (Fig 4). Hypotonic growth media substantially increased zebrafish Matriptase-1a–mediated cleaveage of zebrafish Par2b in HEK293 cells, and increased amounts of processed / active endogenous Matriptase-1 in MCF-10A cells, implicating a conserved regulation of Matriptase activity by ionic strength. *In vivo*, epidermal tumor formation in zebrafish *atp1b1a* mutant embryos suffering from compromised kidney function is fully dependent on Matriptase activity. Unfortunately, due to their small size, it is technically impossible to directly measure the tonicity of the interstitial fluid within zebrafish embryos and their epidermis; yet, systemic tonicity most likely is strongly reduced in *atp1b1a* mutants and in *pax2a*, *lgl2* double morphants, as reflected by the formation of pericardial edema, a characteristic trait for zebrafish mutants with compromised kidney function [61,62]. Accordingly, incubation of *atp1b1a* mutants and *pax2a*, *lgl2* double morphants in isotonic medium not only heals their pericardial defects (thus interstitial tonicity), but also epidermal aggregate formation (Figs 1 and 7). In sum, these data indicate that a hypotonic pericellular environment can promote Matriptase activation and hence, constitutes a risk factor in Matriptase-dependent tumorigenesis.

### Matriptase activity is regulated by the epithelial cell polarity control system affecting its subcellular localization

However, hypotonicity by itself only has very minor oncogenic effects on the embryonic zebrafish epidermis, indicated by very low rates of epidermal aggregate formation and *mmp9* expression in basal keratinocytes of *pax2a* morphant embryos. Rather, hypotonic stress can only induce tumor formation in combination with loss of epithelial polarity in epidermal cells (Fig 7 and [26]). It has been known for decades that carcinogenesis usually results from mutations in more than one gene [63], and disturbed polarity has been reported to contribute to tumorigenesis in several cases [32,33,34,35,36,37]. However, a functional connection between epithelial polarity regulators and Matriptase-1 in the context of carcinogenesis had, at least to our knowledge, not been reported thus far.

We show here that in MCF-10A breast epithelial cells, Matriptase activity is significantly increased upon the loss of Scribble (Fig 4), a regulator of epithelial cell polarity promoting the basolateral domain, like ATP1b and Lgl. This is in line with the increased Matriptase activity formerly reported for breast cancer cells (which have largely lost their epithelial polarity) in comparison to untransformed MCF-10A breast epithelial cells [9], and with our data obtained in zebrafish embryos, where loss of the epithelial cell polarity regulators *atp1b1a* and *lgl2* both promote Matriptase-mediated pre-neoplastic processes (Figs 1 and 7).

Of note, however, loss of *atp1b1a* and *lgl2* in zebrafish does not seem to lead to a general increase of Matriptase-1a protein levels or activity, but to changes in its subcellular localization. In wild-type peridermal cells, it is predominantly localized at lateral domains, whereas it shows a relatively higher abundance in basal domains upon loss of *atp1b1a* or *lgl2* (Fig 6A–6D). It is not clear how epithelial / apico-basal cell polarity contributes to this differential lateral versus basal regulation. With respect to epithelial cell polarity, the bi-layered embryonic zebrafish epidermis constitutes an intermediate between mono-layered simple epithelia and multi-layered stratified epithelia [64,65,66]. Thus, a distinct apical domain and tight junctions are only present in the outer / peridermal cells, and a distinct basal domain with hemidesmosomes adhering to the underlying basement membrane only in basal keratinocytes. All other domains of cells in both layers display similar “lateral-like” properties, characterized by E-cadherin-dependent cell-cell contacts [67]. Yet, even these “lateral-like” domains display a certain epithelial polarity, with highest levels of the cell-cell adhesion molecule E-cadherin levels at the interface of the two cell layers [67]. However, loss of *atp1b1a* or *lgl2* does not affect this E-cadherin polarity [67] or even causes decreased E-cadherin levels [23]. Therefore, the aberrant accumulation of Matriptase-1 in *atp1b1a* mutants on the basal side of peridermal cells most likely is not mediated via E-cadherin [68,69] but might be directly influenced by the disrupted cell polarity.

### Matriptase can act in trans between different cell types and different cell layers to promote tumorigenesis

**Fig 8:**
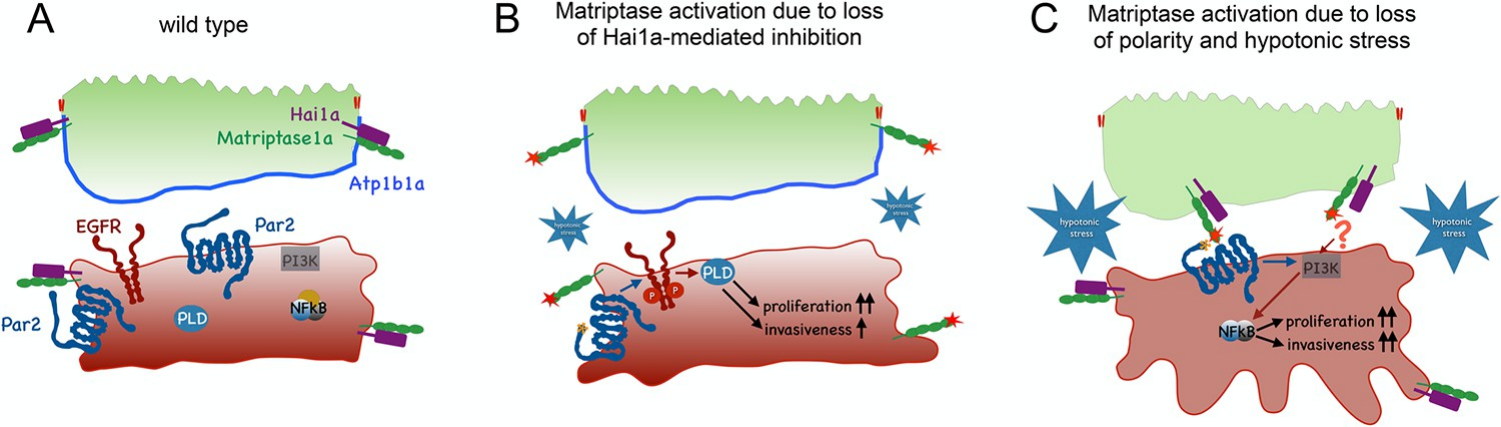
Proposed mechanisms underlying the differential activations and oncogenic activities of Matriptase-1 in *hai1a* and *atp1b1a* mutants to induce cell proliferation, EMT and invasiveness. (A) Model of Matriptase activity restriction in the wild-type zebrafish epidermis. Hai1a is tightly associated with Matriptase, thereby inhibiting its activity, both in peridermal and basal cells. In addition, Matriptase trans-layer signaling from the periderm to the basal layer is restrained by confined levels of Matriptase at the basal side of peridermal cells. (B) Upon loss of Hai1a, Matriptase 1 is no longer inhibited (red star), leading to the cleavage of adjacent Par2b (orange asterisk), which in turn activates the EGFR-PLD pathway, to induce hyperproliferation and expression of *mmp9* (a marker for EMT). Mild hypotonicity (small blue star), most likely due to compromised epidermal integrity, further enhances Matriptase activity levels. Note that additional pathways downstream of Par2b have been described, which lead to additional pre-neoplastic events, like sterile inflammation; however, they do not include PI3K [23]. (C) More extreme hypotonicity in the pericellular space (due to loss of ATP1b1a or Pax2a; large blue star) causes (moderate) Matriptase activation even in the presence of Hai1a. This, however, only has subtle effects on epidermal cells (Fig 7K,7L), unless occurring in conjunction with the loss of epithelial polarity (due to loss of ATP1b1a or Lgl2), allowing Matriptase to shift towards the basal side of peridermal cells, thereby getting into physical contact and to cleave / activate Par2b and other, not yet identified targets (indicated by?) on underlying basal keratinocytes in trans. These targets activate a PI3K-pAKT-mTORC1-NFkB pathway in basal cells resulting in pre-neoplastic events like hyperproliferation, EMT and, in contrast to *hai1a* mutants, strong invasiveness of basal cells.

Strikingly, this shift of Matriptase-1a towards the basal side of peridermal cells of *atp1b1a* mutants might enable exposure of the protease to target substrates on basal cells that are normally constrained from cleavage due to the lack of direct physical contact (Fig 8C). Thus, in contrast to wild-type siblings, in which a transgene-encoded Par2b-GFP fusion protein was almost exclusively found at the cell surface of basal keratinocytes, a large fraction of basal keratinocytes of *atp1b1a* mutants displayed additional, Matriptase-dependent, localization of Par2b-GFP in intracellular vesicle-like structures. This implies that Par2b gets proteolytically activated and internalized in basal keratinocytes by Matriptase (Fig 6E–6H). In addition, our cell autonomy studies with chimeric embryos indicated that Matriptase is required in the peridermal cells to mediate the effect of loss of ATP1b1a function in inducing basal cell transformation (Fig 5). Together, this strongly suggests that in *atp1b1a* mutants, a shift towards the basal side of peridermal cells allows Matriptase to act in trans to cleave substrates such as Par2b on the underlying basal cells (Fig 8). Similar non-cell-autonomous functions of Matriptase have been reported before; for example Matriptase located on thymic epithelial cells can cleave the receptor tyrosine kinase Tie2 on endothelial cells, thereby inducing PI3K signaling in the target cells [70]. Moreover, in a human invasive oral squamous cell carcinoma line, deregulated Matriptase activity due to loss of Hai1 induces Par2 signaling in Par2-expressing cancer-associated fibroblasts, thereby promoting cancer progression [53]. These findings, together with our zebrafish data, show the potential of Matriptase to not only promote tumorigenesis in a cell-autonomous manner, cleaving targets in cis on the tumor cells themselves, but also in a non-cell-autonomous manner, cleaving target proteins on other cells in trans. Of note, the latter can occur in two ways, with Matriptase on tumor cells cleaving targets on the surface of other cells of the tumor microenvironment [53], or with Matriptase from cells of the tumor microenvironment cleaving targets on the prospective tumor cells, as demonstrated here for outer and (pre-neoplastic) basal keratinocytes of the embryonic zebrafish epidermis.

### Aberrant regulation of Matriptase-1 in *hai1a* and *atp1b1a* mutants leads to the activation of different oncogenic pathways and responses

Aberrant Matriptase activity is associated with tumor initiation as well as progression and metastasis in a variety of epithelia-derived cancers [2], mediated by different pathways. For example, in some breast cancers and in human squamous cell carcinomas, Matriptase is responsible for cancer progression by increasing cell proliferation and invasiveness via an HGF (Hepatocyte growth factor)—cMET—pAKT pathway [50,71]. In other breast cancer cell lines, it signals via a Par2—phospholipase C γ2 (PLCγ2)—protein kinase C (PKC) pathway, resulting in the upregulation of NFkB and subsequent *mmp9* expression responsible for metastasis [49].

In zebrafish embryos, deregulated Matriptase due to the loss of Hai1a has previously been shown to act mainly via Par2b, whereas downstream of Par2b, several pathways diverge to lead to different pre-neoplastic events. One of them, an EGFR—Phospholipase D (PLD) pathway, branches off at the level of the PLD product phosphatidic acid and promotes 1) mTORC1 signaling and thereby epidermal cell proliferation and *mmp9* expression, and 2) sphingosine kinase activity and thereby S1P production and apical cell extrusion of epidermal cells [20]. Additionally, Matriptase-activated Par2b in *hai1a* mutants can activate PLC, leading to sterile inflammation via PLC-induced IP3 signaling as well as interference with cell adhesion via PLC-induced DAG-MAPK-RSK signaling [23].

In the *atp1b1a* mutant, however, Matriptase induces an oncogenic pathway that is clearly distinct from those initiated in *hai1a* mutants. Thus, epidermal defects of both *hai1a* and *atp1b1a* mutants are fully dependent on Matriptase-1a, whereas pharmacological inhibition of EGFR and PLD only rescued hyperproliferation and *mmp9* expression in *hai1a*, but not in *atp1b1a* mutants. Conversely, pharmacological inhibition of PI3K and NFkB only rescued *atp1b1a*, but not *hai1a* mutant epidermal defects ([20] and this work). These observations demonstrate that Matriptase has the capacity to induce different downstream pathways also in the zebrafish epidermis. Whether aberrant Matriptase-1a activity in *atp1b1a* also induces PLC-dependent pathways as in *hai1a* mutants [23], and whether these pathways can contribute to the *atp1b1a* mutant phenotype remains to be determined.

### Future perspectives

Future studies also need to elucidate in more detail the molecular mechanisms via which the different modes of aberrant Matriptase-1 regulation lead to the activation of different oncogenic pathways and different cellular responses. Of note, whereas oncogenic effects in the *hai1a* mutant seem to fully depend on the direct Matriptase-1 target Par2b [20,23], loss of *par2b* only partially blocks tumorigenesis in *atp1b1a* mutants. This suggests that in *atp1b1a* mutants, Par2b might act in partial functional redundancy with other Matriptase-1 targets to preferentially promote the oncogenic PI3K-AKT-mTORC1-NFkB pathway. Potentially, these other targets can only be activated in *atp1b1a* mutants as here, disrupted cell polarity might enable their contact with Matriptase-1a, whereas in *hai1a* mutants, in which cells are still polarized, this contact is spatially restricted. Preliminary data obtained via combined rescue experiments of *atp1b1a* mutants point to cMET-related receptor tyrosine kinases [50,71,72] as such potential additional Matriptase-1 targets with partial functional redundancy to Par2b, possibly contributing to the more invasive properties of transformed basal keratinocytes in *atp1b1a* [26] compared to *hai1a* [21] mutants. If so, blockade of these receptor tyrosine kinases should alleviate the highly invasive phenotype of keratinocytes in *atp1b1a* mutants to the milder *“hai1a”*-like phenotype mediated by Par2b only.

Transcriptomics analyses are in progress to further elucidate the oncogenic outcomes downstream of Matriptase and its different pathways in *hai1a* versus *atp1b1a* mutants and their potential impact on the higher aggressiveness of transformed keratinocytes in the *atp1b1a* mutant. Of note, the Matriptase-encoding gene *st14a* is not only expressed in peridermal cells, but also in basal cells themselves [21]. In this light, it will be interesting to investigate whether Matriptase-1 takes over a crucial role in transformed basal keratinocytes themselves during later steps of carcinogenesis, as reported formerly in the context of metastasizing human squamous cell carcinomas [53]. In addition, single cell RNAseq approaches might help to elucidate potential epigenetic heterogeneities [73,74] among the basal keratinocytes in each mutant itself. Indeed, although all cells in the affected relatively young embryos should be genetically identical, epidermal aggregates of *atp1b1a* mutants are formed as discrete cell clusters, and intracellular Par2b, the likely direct result of increased Matriptase activity (Fig 6E–6H), as well as increased NFkB activity downstream of Matriptase (Fig 3G–3I and [26]), were observed in similar patterns. In this light, it is tempting to speculate that differential levels of Matriptase-1 activation might contribute to such intratumoral heterogeneity.

## Methods

### Ethics statement

All zebrafish experiments were approved by the local and federal animal care committees (Landesamt für Natur, Umwelt und Verbraucherschutz (LANUV) Nordrhein-Westfalen: 84–02.04.2012.A251, 84–02.04.2012.A390, 81–02.04.40.2022.VG005, 81–02.04.2018.A281, 81–02.04.2022.A104; City of Cologne: 8.87–50.10.31.08.129).

### Zebrafish lines

Wild-type fish were used from intercrosses of TL and EK. The following transgenic and mutant lines have been described before: *Tg(Ola*.*Actb*:*Hsa*.*hras-egfp)*^*vu119*^ [75], and *Tg(NFκB-RE*:*eGFP)*^*sh235Tg*^ (NFκB responder) [76], *Et(Gal4-VP16)*^*zc1044a*^ [52], *atp1b1a*^*m14*^ [26], *clint1*^*hi1520*^ [22], *hai1a*^*hi2217*^ [21], and *epcam*^*hi2151*^ [42]. The mutant lines *st14a*^*fr56*^, *par2b*^*fr57*^, and the transgenic lines *Tg(uas*:*egfp-st14a)*^*fr58Tg*^ and *Tg(ΔNp63*:*par2b-egfp)*^*fr59Tg*^ were generated in this study.

Embryos were raised in E3 medium (5 mM NaCl, 0.17 mM KCl, 0.33 mM CaCl_2_, 0.33 mM MgSO_4_; hypotonic), or E3 containing 250 mM Mannitol (isotonic).

### Generation and genotyping of *st14a*^*fr56*^ CRISPR/Cas mutant

The guide RNA GGTGATCCTGGCAGCTGTTT targeting exon 3 of *st14a* was designed using the webtool at http://crispr.mit.edu/. The specific Alt-R CRISPR-Cas9 crRNA and the universal Alt-R TM tracrRNA were obtained from IDT, Belgium, and annealed in equal amounts of 30 μM each in Nuclease-Free Duplex Buffer (IDT) by heating to 95°C for 5 min and subsequent cooling to room temperature. 9 μM of complexed RNA was injected together with 150 ng/μl of Cas9 mRNA generated via the SP6 mMessage mMachine kit (Ambion) from plasmid pCS2-Cas9 (gift from Alex Schier; Addgene plasmid # 47322; http://n2t.net/addgene:47322; RRID:Addgene_47322) [77] in Danieau buffer into the zygote. Embryos were raised and their progeny screened for germline transmission by PCR with the primers 5’-TTG ATT TCA GAG AGA CCG GAA T-3‘ and 5’-TCT CCT TAT TTA GAT TAA GGC AAA AC-3‘, followed by a T7 endonuclease assay (NEB) to detect indels and to establish the line *st14a* with a 5 bp deletion in exon 3 (see Results). Genotyping was performed using the same primers followed by *Mwo*I digestion, which results in a cleaved wild-type PCR product and non-cleaved mutant product.

### Generation and genotyping of *par2b*^*fr57*^ CRISPR/Cas mutant

The *par2b* mutant was generated applying the same method, using as guide RNA TGGCGGTGTCCGAGAGCTAC targeting exon 1 of *par2b*. Embryos were raised and their progeny screened for germline transmission by PCR using the primers 5’-CGG CAG AAC TCA ACG CTT C-3‘ and 5’-AGA GCA ACG CAC AAA ACA GG-3‘ followed by a T7 endonuclease assay (NEB) to detect indels and to establish the line *par2* ^***fr57***^ with a 10 bp deletion in exon 1 (see Results). Genotyping was performed using the primer 5’-AGC TGG ATC TGA CTG GAT CG-3‘ in combination with the primer 5’-AAA TCC TGT AGC TCT CGG AC-3‘ to detect the wild-type allele and in combination with the primer 5’-AAT AAT AAA ATC CTG ACA CC-3‘ to detect the mutant allele.

### Generation of *lgl2* CRISPR/Cas9 crispants

The guide RNAs GCTTCACGACGAGAATGCGG and ATGAAAACCCGCTGAACCCG targeting exon 4 and 7 of *lgl2* were designed using the webtool at http://chopchop.cbu.uib.no and obtained as above. 7.5 μM of complexed RNA of both gRNAs were incubated with 10 μg Alt-R S.p. HiFi Cas9 Nuclease V3 (IDT, Belgium) for 5 min at RT and injected either with or without *pax2a* MO into the zygote. To test for efficiency, PCR with the primers 5’-CGT GAT CCA TTA CAC CCC TAT T-3’ and 5’-AAG CC ACA GGA TTA AAA GTC CA-3’ for gRNA #1 and 5’-TTC CCA GAG TTC CAG AGG ATT A-3’ and 5’-GAA ACA TTG CGA AAT ACT GCA C-3’ for gRNA#2 was performed on DNA from 1 dpf embryos, followed by a T7 endonuclease assay (NEB).

### Morpholinos

The following morpholinos were obtained from GeneTools (Philomath, CA), diluted in Danieau buffer to the indicated concentration, and 3 nl were injected into 1-cell stage embryos according to standard protocols.

**Table:** 

Gene	Morpholino sequence	Concentration	Reference
*par2b*	GTAGCTCTCGGACACCGCCATATTC	0.2 mM	[20]
*spint1a*	ACCCTGAGTAGAGCCAGAGTCATCC	0.05 mM	[21]
*atp1b1a*	CGGTATTTAGTTCCCTTTTTGGTGG	0.05 mM	[78]
*lgl2*	GCCCATGACGCCTGAACCTCTTCAT	0.1 mM	[39]
*pax2a*	ATGTGCTTTTTCTTACCTTCCGAGA	0.1 mM	[79]
*st14a*	AACGCATTCCTCCATCCATAGGGTC	0.05 mM	[21]
control	CCTCTTACCTCAGTTACAATTTATA	0–05–0.2 mM	GeneTools

### Quantitative-RT-PCR (RT-qPCR)

Total RNA was isolated from 10–15 embryos at 54 hpf using Trizol (Thermo Fisher Scientific) following standard procedures, followed by a DNaseI treatment (Roche). First-strand cDNA synthesis was performed using reverse transcription (Promega). RT-qPCR was performed in triplicates with Sybr Select Master Mix (Life Technologies, Thermo Fisher Scientific) on an ABI-Prism 7500 Fast Detect system, and relative expression levels were calculated by the ΔΔCt method with *gapdh* as the control gene. Data are presented as fold change relative to the relevant sibling control and represent the average of at least three independent experiments. Primer sequences were as follows: *gapdh* forward 5’-CGC TGG CAT CTC CCT CAA-3’, *gapdh* reverse 5’-TCA GCA ACA CGA TGG CTG TAG-3’ [80], *mmp9* forward 5’-TGA TGT GCT TGG ACC ACG TAA-3’, *mmp9* reverse 5’-ACA GGA GCA CCT TGC CTT TTC-3’ [81], *hai1a* forward 5’-GGA GCA CAG AGA AGA TCC TA-3’, and *hai1a* reverse 5’-CGT GGA GGT CTA TCC TCT ACA T-3’ [20] and *st14a* forward 5’-ATC TTC TCA TCT CAC AGA AGT GG-3’ and *st14a* reverse 5’-GCT TGG TCC CAG TCC TTG TC-3’.

### Plasmid generation and injection, and generation of transgenic lines

The construct *uas*:*egfp-st14a* was generated using the Tol2 kit [82], with the described *uas* promoter in the p5E vector, the *egfp* sequence in the pME vector, and the *st14a* cDNA sequence amplified with the following primers: 5’-GGG GAC AGC TTT CTT GTA CAA AGT GGC CAT GGA CCC TAT GGA TGG AGG A-3’ and 5’-GGG GAC AAC TTT GTA TAA TAA AGT TGC TTA CAC TCC CGT CTT CTC CTT G-3’ in p3E. The obtained plasmid was injected to generate the stable transgenic line *Tg(uas*:*gfp-st14a)*^*fr58Tg*^ that was used in conjunction with the periderm-specific driver *Et(Gal4-VP16)*^*zc1044a*^ [52] (*peri*:*Gal4*; Fig 5) for exclusive expression of GFP-labelled Matriptase-1 in the outer epidermal cell layer. For mosaic expression of *egfp-st14a* in peridermal cells (Fig 6), a *krt4*:*egfp-st14a* construct was obtained as above, with the described *krt4* promoter [83] instead of *uas* and injected into 1-cell stage wild-type embryos at a concentration of 10 ng/μl. Embryos were analysed at 48 hpf using confocal microscopy. The construct *ΔNp63*:*par2b-egfp* was generated by first using the Tol2 kit to C-terminally fuse the *par2b* coding sequence with *egfp* (the following primers were used to amplify *par2b* cDNA for insertion into pME: 5’-GGG GAC AAG TTT GAC AAA AAA GCA GGC TCC ACC ATG GCG GTG TCC GAG AGC TA-3’ and 5’-GGG GAC CAC TTT GTA CAA GAA AGC TGG GTA TCA GCA AGT GCT GGT TTC CG-3’). Subsequently, *parb2-egfp* was amplified using the primers 5’-GAA GGA TAT CTC CAC CAT GGC GGT GTC CGA-3’ and 5’-CTT CGA TAT CTC CCT ATA GGG CTG CAG AAT CTA-3’, digested by *Eco*RV, and cloned into the *Eco*RV site of the modified pT2AL200R150G vector containing the *ΔNp63* promoter [20]. The obtained plasmid was used to obtain the line *Tg(ΔNp63*:*par2b-egfp)*^*fr59Tg*^ by standard injection and screening procedures, which was then used to analyse Par2b-eGFP subcellular localization.

### Cell transplantations

Ventral ectodermal cells from *Tg(Ola*.*Actb*:*Hsa*.*hras-egfp)*^*vu119*^ transgenic donor embryos, either injected with *atp1b1a* MO or *atp1b1a* MO and *st14a* MO were transplanted at 6 hpf into the ventral ectoderm of *atp1b1a*, *st14a* double morphant or *atp1b1a* single morphant recipients. Chimeric recipients were raised in E3 embryo medium and fixed at 58 hpf.

### Whole-mount *in situ* hybridization (WISH) and colorimetric immunostainings

Embryos were fixed in 4% paraformaldehyde (PFA) and WISH was performed as previously described [84]. *mmp9* cDNA templates and DIG-labeled probes were synthesized with the Roche digoxygenin RNA synthesis kit, as described [21,38]. Combined colorimetric WISH and immunostainings were performed as described [21] using the primary antibodies mouse anti-GFP (Invitrogen; A10262, 1:300), or mouse anti-Tp63 BC4A4 (Zytomed, 1:200), and the secondary biotinylated horse anti-mouse IgG (Vector Laboratories). ABC amplification was performed using the Vectastain Elite ABC Peroxidase kit (Vector Laboratories), and embryos were incubated in DAB substrate (Sigma-Aldrich) and H_2_O_2_ until development of a signal.

### Immunofluorescence (IF) analyses

To determine cell proliferation, embryos were incubated in 10mM BrdU in E3 for 1 hour at 4°C, followed by a one-hour wash with E3 at 28C and fixation in 4% PFA. BrdU incorporation was detected by anti-BrdU immunolabelling. IF analyses were performed essentially as previously described [21]. Embryos were fixed in 4% PFA for stainings using the following primary antibodies: mouse anti-BrdU (Roche 1170376, 1∶100), mouse anti-Tjp1 (Zymed; 33–9100, 1:200), chicken anti-GFP (Invitrogen; A10262, 1:300), mouse anti-p63 BC4A4 (Zytomed, 1:200), rabbit anti-zebrafish Lgl2 [85] (1:400), rabbit anti-aPKC (C-20, Santa Cruz sc-216, 1:200), rabbit anti-phospho- ribosomal protein S6 (pRPS6, Ser240/241; Cell Signaling, #2215, 1∶300) and mouse anti-chicken ATPa6F (Developmental Studies Hybridoma Bank; DSHB).

For stainings with the mouse anti-panKeratin1-8 (Progen 61006, 1:10), embryos were fixed with Dent’s fixative (80% Methanol, 20% DMSO, at -20°C overnight). For pAKT stainings, embryos were fixed in EAF (40% ethanol, 5% acetic acid, 4% formaldehyde in PBS) and either washed with PBS-TritonX100, followed by an antigen retrieval in 10 mM Tris, 1 mM EDTA, pH 9.0 for 60 min at 60°C, blocking in 5% sheep serum and antibody incubation with rabbit anti-pAKT (S473) (Cell signaling #4060, 1:50) or processed for cryosectioning as described [86]. Secondary antibodies were anti-mouseCy3, anti-rabbitCy3, anti-mouseAlexa488, and anti-chickenAlexa488 (all Invitrogen, 1:500).

### Inhibitor treatments

Wortmannin (Sigma-Aldrich), LY294002 (TocrisBioscience), and Rapamycin (Merck), were dissolved in DMSO, and inhibitors were diluted in E3 embryo medium to concentrations of 1 μM (Wortmannin), 25 μM (LY294002), or 1.1 μM (Rapamycin), Embryos were incubated in inhibitor solution starting from 34 hpf and analyzed or fixed in 4% PFA at 54 hpf.

### Microscopy

Images were taken using a Zeiss Confocal (LSM710 META), an AxioImager microscope (Zeiss) equipped with an Apotome, using the AxioVision software (Zeiss), an Axioplan2 microscope (Zeiss) using AxioVision software (Zeiss), or a Leica M165FC stereo microscope with DFC425C camera and the Leica Application Suite V3.8 software. Images were processed using the ImageJ software and Adobe Photoshop.

### HEK293 cell culture and Alkaline Phosphatase (AP) release assay

Plasmids for the zebrafish Matriptase activity test were generated as follows: for pcDNA3.1+st14a, the *st14a* cDNA was amplified using the oligos 5’-GGC CGG ATC CAC CAT GGA CCC TAT GGA TGG AGG AAT-3’ and 5’-GGC CGC GGC CGC TTA CAC TCC CGT CTT CTC CTT-3’, cloned into the *Eco*RV site of pBluescript SK(-) (Addgene), and from the obtained plasmid, a *Kpn*I—*Not*I fragment containing the *st14a* cDNA was subcloned between the *Kpn*I and *Not*I sites of pcDNA3.1. To obtain pcDNA3+AP-par2b, the *SEAP* coding sequence was cut out of pCMV-SEAP (Addgene) with *Eco*RI and *Xba*I and inserted into the *Eco*RI and *Xba*I sites of pCDNA3.1. Subsequently, the *par2b* coding sequence was amplified using the primers 5’-GGA GGC CTC CGA CTA CAA AGA CGA TGA CGA CAA GGA CGC CCA GCC AGG CAA AAA TGG-3’ and 5’-GGC CGT TAA CTC AGC AAG TGC TGG TTT CCG TGT T-3’ adding a flag tag as linker sequence between SEAP and aa21 of Par2b, and this PCR product was cut with *Stu*I and *Hpa*I and cloned into the *Hpa*I site at the 3’ end of SEAP, removing its stop codon.

The AP release assay was done as described by [25] with slight modification. Briefly, HEK 293T cells were plated in DMEM (Gibco) with 10% FBS at a density of 0.8 × 10^5^ cells/well of a 24-well plate. Approximately 24 h later, cells were left untransfected, or transfected by using Lipofectamine 3000 reagent (Invitrogen) with 500 ng pcDNA3.1-AP-Par2b and 4 ng empty pcDNA3.1 or 500 ng pcDNA3.1-AP-Par2b and 4 ng pcDNA3.1-st14a. Approximately 24 hours later, wells were washed once with 0.5 ml DMEM medium containing 0.1% BSA and 20 mM Hepes, and 300 μl medium with different osmolarity was added to each well. The medium with isotonicity (320 mOsmol/l was prepared by adding 20% FBS to DMEM and diluted 1 to 1 with 150 mM Na-Glutamate. Media diluted 1 to 1 with 110 mM, 70 mM and 0 mM Na-Glutamate and measured by osmometer have an osmolarity of approximately 270, 230 and 150 mOsmol/l respectively. After being incubated for 45 min at 37°C, 200 μl medium was removed and transferred to labeled tubes kept on ice. The samples were centrifuged at 13,000 rpm for 5 min, and 25 μl of each sample was added to 50 μl of assay buffer from the NovaBright Phospha-Light EXP Assay Kit (Invitrogen) and heated for 5 min at 65°C. Samples were then cooled to room temperature, and moved into a 96-well plate. 50 μl reaction buffer containing AP chemiluminescent substrate from the kit was added to each well and incubated for 20 min. Chemiluminescence was measured in a microplate luminometer (Infinite 200 Pro, Tecan). All samples were in triplicate.

### MCF10A culture and hypotonicity assays

The human breast epithelial cell line MCF10A was cultivated at 37°C with 5% CO_2_ and grown to about 80% confluency before passaging. MCF10A cells were maintained in DMEM:F12 with GlutaMAX (Life Technologies), supplemented with 5% donor horse serum, 20 ng/ml EGF, 100 ng/ml cholera toxin, 0.5 μg/ml hydrocortisone, 10 μg/ml insulin, 1x Pen/strep, 15 mM HEPES. Media with different osmolalities were prepared by diluting MCF10A medium supplemented with 10% (instead of 5%) horse serum: 320 mOsmol/l (isotonic): 1:1 dilution of 160 mM mono-sodium glutamate (MSG; Sigma-Aldrich) with MCF10A medium (10% horse serum); 230 mOsmol/l (moderate hypotonic): 1:1 dilution of 70 mM MSG with MCF10A medium (10% horse serum); 150 mOsmol/l (strong hypotonic): 1:1 dilution of H_2_O dest. with MCF10A medium (10% horse serum).

### Generation of SCRIB knockout and rescue MCF10A cells

Scribble KO cells were engineered using the CRISPR/Cas9 gene editing approach with guide arms targeting Scribble’s exon 1 (5’-GAA GCG GCA CTG TTC GCT GC-3’). Scribble guide arms were cloned into the pLX-sgRNA vector (Addgene #50662) and transfected into 293T cells along with the pCW-Cas9 vector (Addgene #50661) and lentiviral packaging vectors to produce lentivirus for co-transduction into MCF10A cells. Positive cells were treated using 2 μg/mL puromycin for pCW-Cas9 and 5 μg/mL blasticidin for pLX-sgRNA selection for 7 days. Following antibiotic selection, pCW-Cas9 only and Scribble KO (pCW-Cas9 and pLX-sgRNA containing) clonal cell lines were generated using serial dilution to isolate colonies. Scribble KO for each clone was confirmed using western blot and immunofluorescence microscopy. Full length SCRIB was cloned into a retroviral murine stem cell virus (MSCV) backbone containing a bicistronic internal ribosome entry site (IRES) for stable expression in MCF10A cells.

### SDS-PAGE and western blot analyses

SDS-PAGE and immunoblot analysis was performed as previously described [87]. Briefly, cells were seeded in 6-well plates, kept in isotonic or hypotonic medium for 24 hrs as indicated, followed by PBS wash and total lysis using hot crude lysis buffer (1% SDS, 10 mM EDTA). After lysate processing and protein measurement, samples were separated on self-cast SDS-PAGE and transferred to PVDF membranes by wet-blot transfer (Invitrogen) for subsequent immunoblotting. 5% BSA/TBST was used for blocking unspecific binding sites as well as for primary and secondary antibody incubations. The following antibodies were used: mouse anti-GAPDH (Millipore, MAB374, 1:10000), goat anti-Hai1 (R&D Systems, AF1048-SP, 1 ug/ml), rabbit anti-Matriptase/MT-SP1 (Sigma Aldrich, IM1014-50UG, 1:1000), mouse anti-Scribble ([88], clone 7C6.D10, 1:100), sheep anti-mouse HRP (Amersham, NA931V, 1:4000), donkey anti-rabbit HRP (Amersham, NA9340V, 1:4000), and rabbit anti-goat HRP (Invitrogen, 81–1620, 1:4000). Following enhanced chemiluminescence reaction, resulting western blot signals were visualized and quantified using a digital ImageQuant 800 Detector system (Cytiva).

### Statistics

Quantitative experiments were repeated at least three times, reaching similar results. Mean values and standard deviations of all individual specimens (biological samples; n) from one representative or all independent experiments (N) are presented, as specified in the respective figure legends. Statistical analysis was performed using Graph Pad Prism software. For comparison of multiple groups, one-way ANOVA with post-hoc Tukey’s test was used; for comparison of two groups, an unpaired two-tailed Student’s t-test was used to determine significance, for comparison of distributions of phenotypes, a Chi-square test was used. Obtained p-values are mentioned in the respective figure legends and provided in S1 Appendix.

## Supporting information

S1 Fig *st14a* is required downstream of *atp1b1a* for aggregate formation in the epidermis. (A-C) Generation of *st14a* CRISPR/Cas9 mutant with 5 bp deletion causing a frame shift and premature stop codon in the transmembrane domain of Matriptase-1. (A) Schematic representation of the *st14a* transcript (ENSDART00000086952.7). (B) Alignment of DNA sequence of exon 3 of wild-type *st14a* and *st14a*^*fr56*^, the latter with a 5 bp deletion in exon 3. The sequence chosen for the CRISPR guide RNA is highlighted in red. (C) Amino acid (AA) sequence of the wild-type and mutant protein (predicted AA sequence resulting from the frame shift is indicated in red). The AA sequence of the transmembrane domain is boxed in yellow, the SEA domain in grey; the CUB domains are indicated by blue boxes, the LDLa domains by green boxes, and the protease domain by an orange box. (D-J) The epidermal aggregate phenotype of *atp1b1a* mutants and *hai1a* morphants is rescued by the *st14a*^*fr56*^ allele. Embryos were obtained from an incross of *atp1b1a*^*m14/+*^, *st14a*^*fr56/+*^ parents or from an incross of *st14a*^*fr56/+*^ parents and injection with a *hai1a* MO. Embryos were phenotyped for epidermal aggregates and subsequently genotyped. (D,E) Bright field images with lateral views of yolk sac / yolk extension region of 58 hpf *atp1b1a-/-*, *st14a*+/+ embryo displaying epidermal aggregates (D; indicated with arrows) and of *atp1b1a-/-*, *st14-/-* sibling, in which aggregates have not formed (E). (F,G). Bright field images with lateral view of yolk sac / yolk extension region of a 24 hpf *hai1a* MO, *st14a*+/+ embryo displaying epidermal aggregates (F; indicated with arrows) and a *hai1a* MO, *st14a-/-* embryo, in which aggregates have not formed (G). H. Image of an ethidium bromide agarose gel showing PCR fragments / *st14a* genotyping results for embryos without (indicated by *) or with aggregates, obtained from *atp1b1a*^*m14/+*^, *st14a*^*fr56/+*^ incross (left panel) or from *st14a*^*fr56/+*^ incross and *hai1a* MO injection (right panel). (I) Bright field images of different severities of the *atp1b1a* mutant phenotype at 56 hpf. (K) Quantification of phenotypes of *atp1b1a* mutants as shown in (D,E, I) and Fig 1I–1N (n = 45–72 embryos from N = 3 independent clutches per condition). (K) Quantification of phenotypes of *hai1a* morphants as shown in (F,G) (n = 66–135 embryos from N = 3 independent clutches per condition, Significances were determined via a Chi-square test, ns, not significantly different (p>0.05); ****, significantly different (p< 0.0001)).
(TIF)

S2 Fig *par2b* is partially required downstream of *ap1b1a* for aggregate formation and increased *mmp9* expression in the epidermis. (A-D) *par2b* MO attenuates the severity of epidermal aggregate formation in *atp1b1a* mutants. Brightfield images of live 58 hpf *atp1b1a* mutant embryos injected with control (A) or *par2b* MO (B-D) displaying different degrees of aggregate formation in the epidermis (A’-D’ for magnified views of the posterior half (yolk extension and tail fin region) of embryos shown in A-D). (E) Quantification of the phenotypic strengths of embryos deriving from *atp1b1a*^*m14/+*^ incross and injected with *par2b* MO (bars 1–2; N = 3 clutches; n = 86 embryos) or from *atp1b1a*^*m14/+*^, *par2b*^*fr57/+*^ incross (bars 3–8; N = 3 clutches; n = 214 embryos; Significances were determined via a Chi-square test, ns, not significantly different (p>0.05), **,***,****, significantly different (p<0.01, 0.001, 0.0001, respectively). Embryos were phenotypically categorized at 58 hpf, followed by genotyping of categorized individuals. (F-H) Representative images of WISH of *mmp9* (blue) in 72 hpf *atp1b1a-/-; par2b+/+* (F), *atp1b1a-/-; par2b+/-* (G), and *atp1b1a-/-; par2b-/-* (H). Compared to the strong *mmp9* expression observed in 100% of the *atp1b1a* single mutant embryos, 30% of *atp1b1a* mutants heterozygous for *par2b* showed a decreased *mmp9* expression. Of the *atp1b1a-/-; par2b-/-* double mutants, 50% did not show obvious *mmp9* staining, comparable to wild type controls (compare with Fig 2M), and the other 50% showed weak *mmp9* staining intensity comparable to that shown in panel (G). (I) RT-qPCR showing relative quantities of *mmp9* transcript of 58 hpf *atp1b1a-/; par2b+/+* mutants compared to their wild-type *atp1b1a; par2b+/+* siblings, and *atp1b1a-/-; par2b-/-* double mutants compared to their wild-type *atp1b1a*, *par2b-/-* siblings (N = 3, n = 15; significances were determined via a one-way ANOVA and Tukey’s post hoc test; ns, not significantly different (p>0.05); ****, significantly different (p<0.0001). (J) Schematic representation of the *par2b* transcript (ENSDART00000114982.4). (K) Alignment of DNA sequence of exon 1 of wild-type *par2b* and *par2b*^*fr57*^ showing a 10 bp deletion in the mutant allele. The sequence chosen for the CRISPR guide RNA is highlighted in red. (L) Amino acid (AA) sequence of the wild-type and mutant protein (predicted AA sequence resulting from the frame shift is indicated in red). The arrowhead indicates the putative Matriptase-1 cleavage site, the blue box marks AAs coding for the tethered ligand, yellow boxes mark AAs coding for the transmembrane domains. (M-Q) The *par2b*^*fr57*^ mutation is able to suppress even more efficiently the epidermal phenotype caused by loss of *hai1a*. (M-P) Brightfield images of tails of live 48 hpf embryos obtained from an incross of *hai1a*^*hi2217/+*^; *par2b*^*fr57*/+^ parents. Images were categorized as wild type-appearing, mild aggregate formation, moderate aggregate formation, or severe aggregate formation, followed by genotyping of categorized individuals. (Q) Quantification of epidermal phenotypes; N = 3 clutches, n = 134 embryos, Significances were determined via a Chi-square test, ns, not significantly different (p>0.05), ****, significantly different (p<0.0001).
(TIF)

S3 Fig Increased AP release upon hypotonicity is dependent on Matriptase. Reporter assay for Matriptase activity towards Par2 cleavage showing that increased AP release at low osmolalities is dependent on Matriptase. HEK293 cells were transfected with pcDNA3+AP-Par2b, and pcDNA3+AP-Par2b together with pcDNA3+St14a. After 24 hrs in regular / isotonic medium (tonicity of 320 mOsm), cells were exposed to media of 320 mOsm and 150 mOsm for 15 (A, B, C) and 45 (D, E, F) minutes, respectively. (A,D) Absolute luminescence values of AP released into the supernatant after 15 (A) and 45 minutes (D), respectively, are highest at 150 mOsm when cells were transfected with both pcDNA3+AP-Par2b and pcDNA3+St14a. (B,E) Subtraction of luminescence values of 320 mOsm from 150 mOsm indicate a significantly higher net increase of AP release in double transfected cells compared to cells transfected with pcDNA3+AP-Par2b alone. (C,F) Percentage of increased AP release upon exposure to 150 mOsm dependent on Matriptase1a was calculated by subtracting the net AP values of AP-Par2b from AP-Par2b/St14a and determining their percentage of the corresponding net AP values from AP-Par2b/St14a. n = 5, different shapes of data points indicate respective experiments, significances were determined via a Student’s t-test.
(TIF)

S4 Fig Knockout of *lgl2* by Crispr/Cas9 in F0 mutants (crispants) phenocopies the effect of *lgl2* morpholino knockdown to induce pre-neoplastic transformations of basal keratinocytes in combination with knockdown of *pax2a*. (A) Schematic representation of the *lgl2* transcript (ENSDARG00000023920). Arrows indicate the locations targeted by Crispr guide RNAs. (B-C). Images of ethidium bromide agarose gels showing PCR fragments obtained from DNA of single embryos and digested by T7 Endonuclease I. PCR fragments from embryos injected with both gRNA#1 and #2 (*) are cut compared to non-injected controls (no *), indicative of occurring indels. (D-F) Immunofluorescence for Lgl2 (red) and p63 (green) on the trunk region of whole mount 54 hpf embryos. Lgl2 is present at cell borders of basal cells in wt embryos (D) but absent from *lgl2* morphants (E) and *lgl2* crispants (F), revealing efficient disruption of the *lgl2* gene (n = 8–10) Scale bar: 20 μm. (G-I) Brightfield images of a live 6 dpf wt embryo (G, G’), *lgl2* morphant (H, H’), and *lgl2* crispant (I, I’); magnified views of tail regions (G’-I’). Whereas the transient knockdown of *lgl2* by morpholinos is not able to induce late epidermal defects, F0 Crispr/Cas9 mutants develop epidermal phenotypes starting at 5–6 dpf in about 60% of injected embryos (N = 3, n = 32–40), reminiscent of the *lgl2/penner* mutant (39, Fig 2B) and demonstrating loss of *lgl2* function. (J-L) Brightfield images of live 54 hpf embryos crispant for *lgl2* to induce polarity defects, morphant for *pax2a* to induce hypotonic stress, or both; overviews of entire embryos (J-L) and magnified views of tail regions (J’-L’). Whereas *lgl2* knockout by Crispr/Cas9 alone does not result in epidermal defects at early stages, it causes epidermal aggregate formation when combined with the knockdown of *pax2a* by morpholinos (L,L’; indicated in L’ with arrows) comparable to the knockdown of *lgl2* by morpholinos (Fig 7A–7C, 7K), Scale bar: 500 μm in overview, 100 μm in magnified image. (K) Quantification of phenotypes of embryos with different genotypes / morphant conditions (N = 3, n = 27–53). Significances were determined via a one-way ANOVA and Tukey’s post hoc test by comparing fraction of embryos displaying an epidermal phenotype (mild, medium, and strong) with no epidermal phenotype; columns with same superscript letter (a,b,c) are not significantly different (p>0.05).
(TIF)

S1 Appendix Numerical data and statistical tests. (XLSX)
