# Supplementary figures and images for: Matriptase-dependent epidermal pre-neoplasm in zebrafish embryos caused by a combination of hypotonic stress and epithelial polarity defects
Source: PLoS Genet. 2023 Aug 11;19(8):e1010873. doi: 10.1371/journal.pgen.1010873 (PMC10446194; doi:10.1371/journal.pgen.1010873)

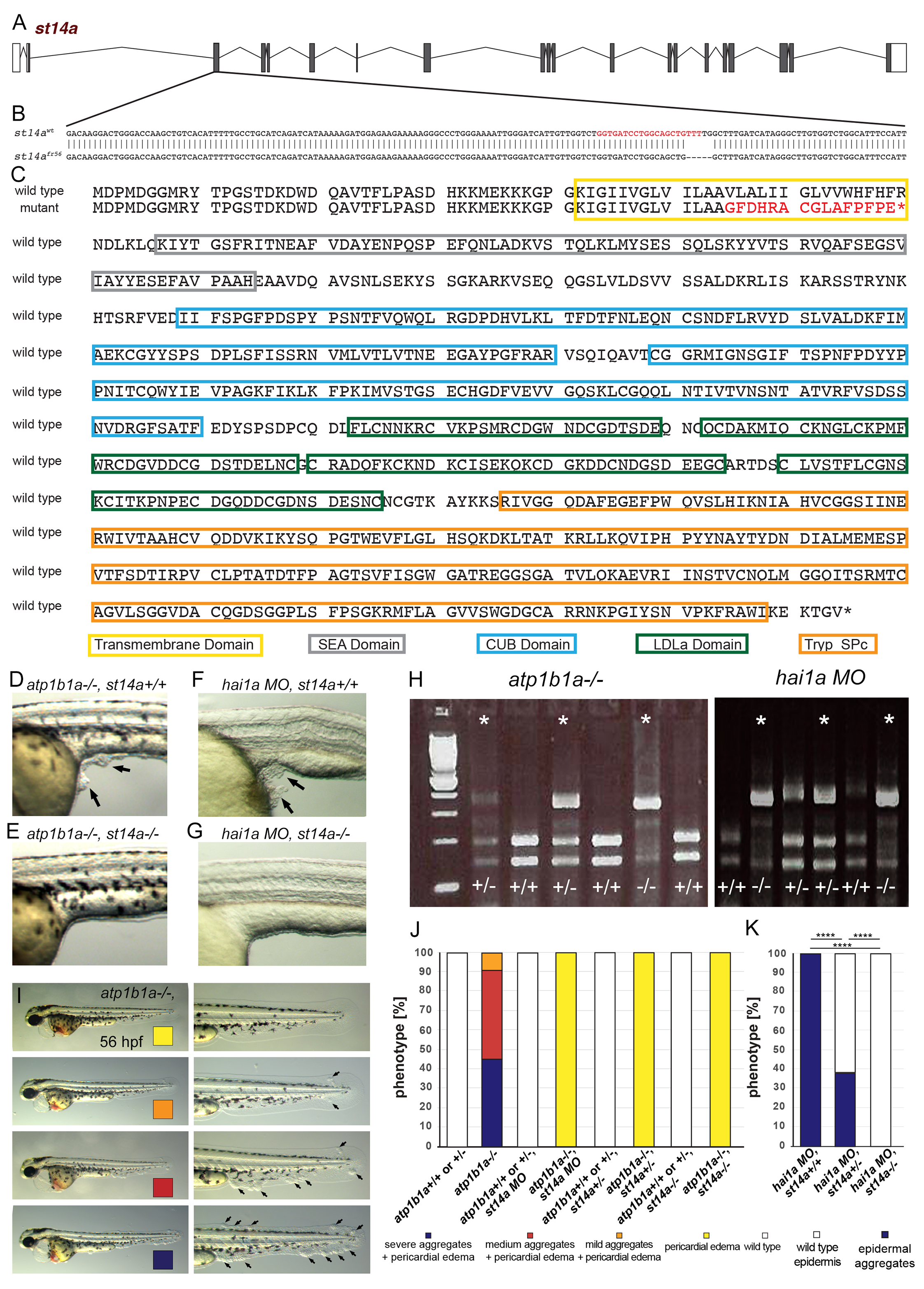

Supplement: S1 Fig — (A-C) Generation of st14a CRISPR/Cas9 mutant with 5 bp deletion causing a frame shift and premature stop codon in the transmembrane domain of Matriptase-1. (A) Schematic representation of the st14a transcript (ENSDART00000086952.7). (B) Alignment of DNA sequence of exon 3 of wild-type st14a and st14afr56, the latter with a 5 bp deletion in exon 3. The sequence chosen for the CRISPR guide RNA is highlighted in red. (C) Amino acid (AA) sequence of the wild-type and mutant protein (predicted AA sequence resulting from the frame shift is indicated in red). The AA sequence of the transmembrane domain is boxed in yellow, the SEA domain in grey; the CUB domains are indicated by blue boxes, the LDLa domains by green boxes, and the protease domain by an orange box. (D-J) The epidermal aggregate phenotype of atp1b1a mutants and hai1a morphants is rescued by the st14afr56 allele. Embryos were obtained from an incross of atp1b1am14/+, st14afr56/+ parents or from an incross of st14afr56/+ parents and injection with a hai1a MO. Embryos were phenotyped for epidermal aggregates and subsequently genotyped. (D,E) Bright field images with lateral views of yolk sac / yolk extension region of 58 hpf atp1b1a-/-, st14a+/+ embryo displaying epidermal aggregates (D; indicated with arrows) and of atp1b1a-/-, st14-/- sibling, in which aggregates have not formed (E). (F,G). Bright field images with lateral view of yolk sac / yolk extension region of a 24 hpf hai1a MO, st14a+/+ embryo displaying epidermal aggregates (F; indicated with arrows) and a hai1a MO, st14a-/- embryo, in which aggregates have not formed (G). H. Image of an ethidium bromide agarose gel showing PCR fragments / st14a genotyping results for embryos without (indicated by *) or with aggregates, obtained from atp1b1am14/+, st14afr56/+ incross (left panel) or from st14afr56/+ incross and hai1a MO injection (right panel). (I) Bright field images of different severities of the atp1b1a mutant phenotype at 56 hpf. (K) Quantification [file pgen.1010873.s001.tif]

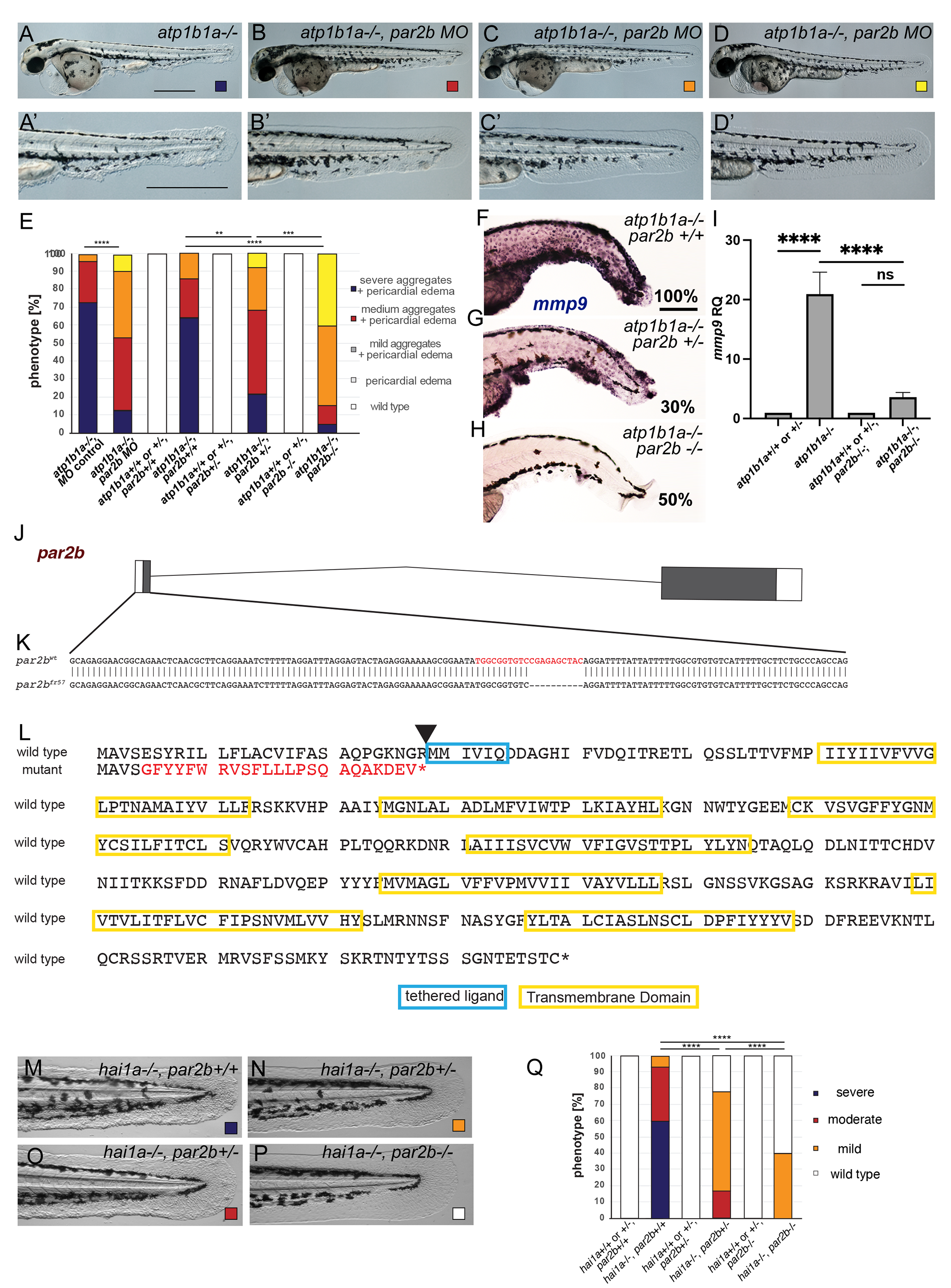

Supplement: S2 Fig — (A-D) par2b MO attenuates the severity of epidermal aggregate formation in atp1b1a mutants. Brightfield images of live 58 hpf atp1b1a mutant embryos injected with control (A) or par2b MO (B-D) displaying different degrees of aggregate formation in the epidermis (A’-D’ for magnified views of the posterior half (yolk extension and tail fin region) of embryos shown in A-D). (E) Quantification of the phenotypic strengths of embryos deriving from atp1b1am14/+ incross and injected with par2b MO (bars 1–2; N = 3 clutches; n = 86 embryos) or from atp1b1am14/+, par2bfr57/+ incross (bars 3–8; N = 3 clutches; n = 214 embryos; Significances were determined via a Chi-square test, ns, not significantly different (p>0.05), **,***,****, significantly different (p<0.01, 0.001, 0.0001, respectively). Embryos were phenotypically categorized at 58 hpf, followed by genotyping of categorized individuals. (F-H) Representative images of WISH of mmp9 (blue) in 72 hpf atp1b1a-/-; par2b+/+ (F), atp1b1a-/-; par2b+/- (G), and atp1b1a-/-; par2b-/- (H). Compared to the strong mmp9 expression observed in 100% of the atp1b1a single mutant embryos, 30% of atp1b1a mutants heterozygous for par2b showed a decreased mmp9 expression. Of the atp1b1a-/-; par2b-/- double mutants, 50% did not show obvious mmp9 staining, comparable to wild type controls (compare with Fig 2M), and the other 50% showed weak mmp9 staining intensity comparable to that shown in panel (G). (I) RT-qPCR showing relative quantities of mmp9 transcript of 58 hpf atp1b1a-/; par2b+/+ mutants compared to their wild-type atp1b1a; par2b+/+ siblings, and atp1b1a-/-; par2b-/- double mutants compared to their wild-type atp1b1a, par2b-/- siblings (N = 3, n = 15; significances were determined via a one-way ANOVA and Tukey’s post hoc test; ns, not significantly different (p>0.05); ****, significantly different (p<0.0001). (J) Schematic representation of the par2b transcript (ENSDART00000114982.4). (K) Alignment of DNA sequence of exon 1 of wild-ty [file pgen.1010873.s002.tif]

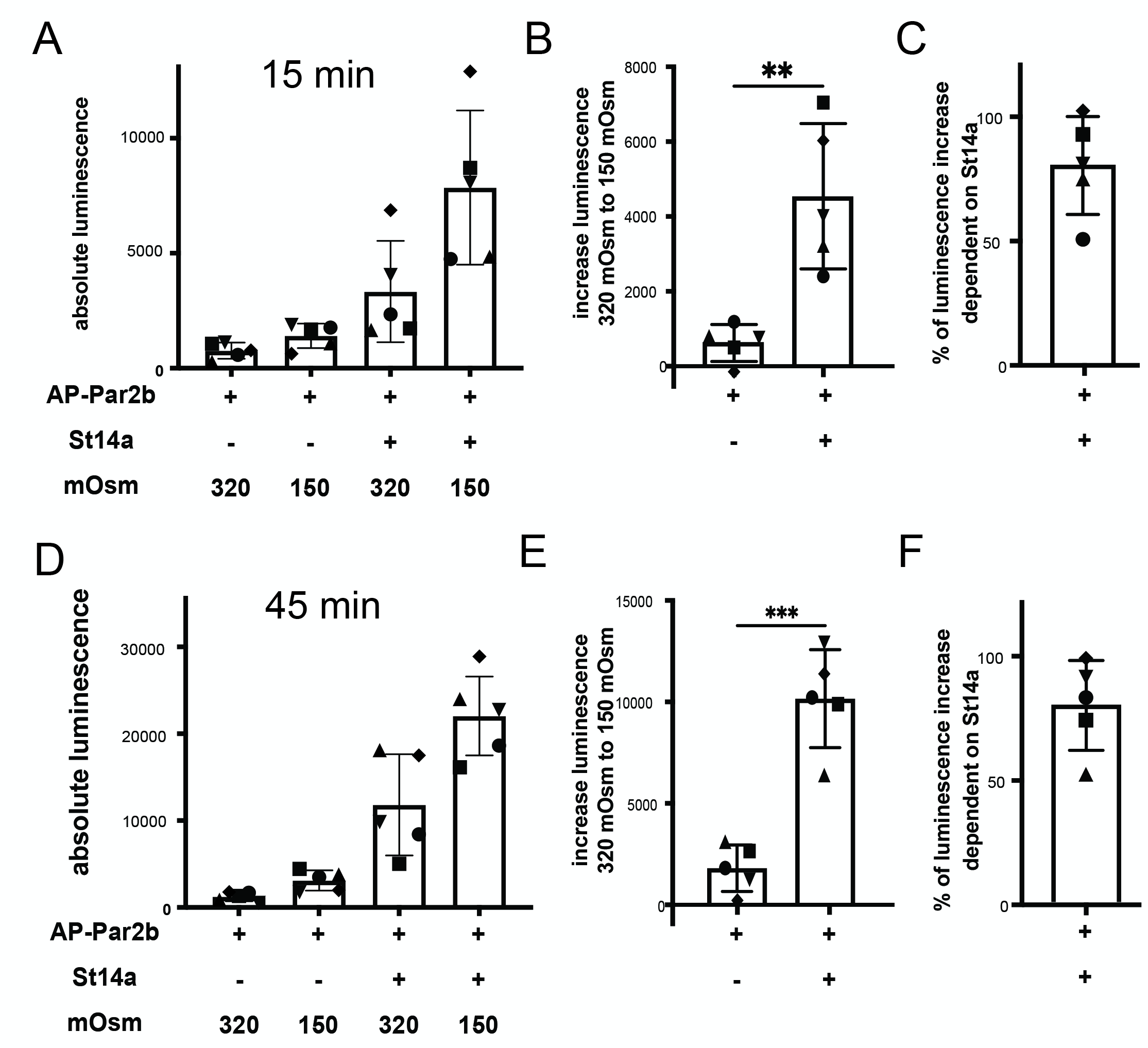

Supplement: S3 Fig — Reporter assay for Matriptase activity towards Par2 cleavage showing that increased AP release at low osmolalities is dependent on Matriptase. HEK293 cells were transfected with pcDNA3+AP-Par2b, and pcDNA3+AP-Par2b together with pcDNA3+St14a. After 24 hrs in regular / isotonic medium (tonicity of 320 mOsm), cells were exposed to media of 320 mOsm and 150 mOsm for 15 (A, B, C) and 45 (D, E, F) minutes, respectively. (A,D) Absolute luminescence values of AP released into the supernatant after 15 (A) and 45 minutes (D), respectively, are highest at 150 mOsm when cells were transfected with both pcDNA3+AP-Par2b and pcDNA3+St14a. (B,E) Subtraction of luminescence values of 320 mOsm from 150 mOsm indicate a significantly higher net increase of AP release in double transfected cells compared to cells transfected with pcDNA3+AP-Par2b alone. (C,F) Percentage of increased AP release upon exposure to 150 mOsm dependent on Matriptase1a was calculated by subtracting the net AP values of AP-Par2b from AP-Par2b/St14a and determining their percentage of the corresponding net AP values from AP-Par2b/St14a. n = 5, different shapes of data points indicate respective experiments, significances were determined via a Student’s t-test. (TIF) [file pgen.1010873.s003.tif]

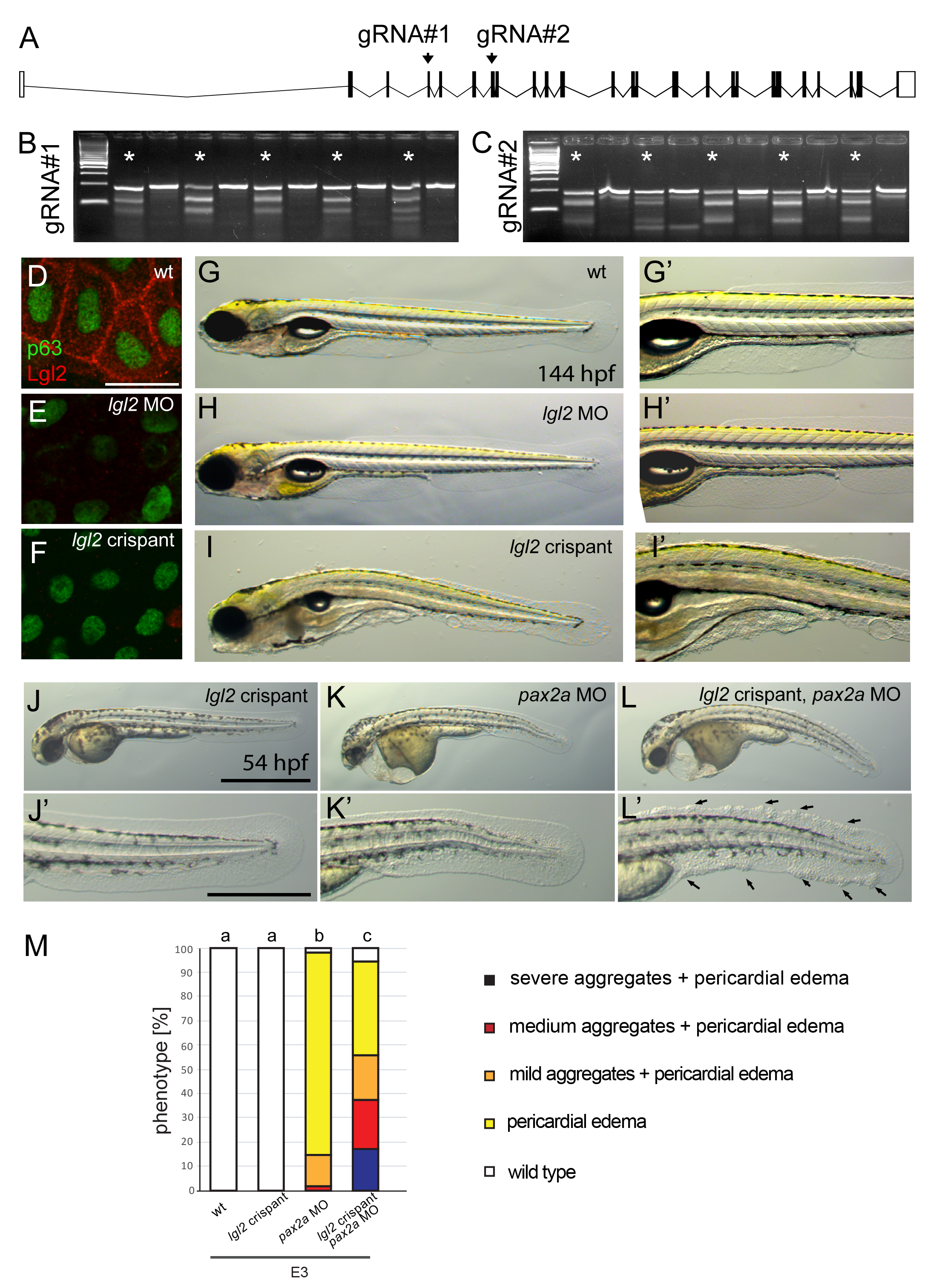

Supplement: S4 Fig — (A) Schematic representation of the lgl2 transcript (ENSDARG00000023920). Arrows indicate the locations targeted by Crispr guide RNAs. (B-C). Images of ethidium bromide agarose gels showing PCR fragments obtained from DNA of single embryos and digested by T7 Endonuclease I. PCR fragments from embryos injected with both gRNA#1 and #2 (*) are cut compared to non-injected controls (no *), indicative of occurring indels. (D-F) Immunofluorescence for Lgl2 (red) and p63 (green) on the trunk region of whole mount 54 hpf embryos. Lgl2 is present at cell borders of basal cells in wt embryos (D) but absent from lgl2 morphants (E) and lgl2 crispants (F), revealing efficient disruption of the lgl2 gene (n = 8–10) Scale bar: 20 μm. (G-I) Brightfield images of a live 6 dpf wt embryo (G, G’), lgl2 morphant (H, H’), and lgl2 crispant (I, I’); magnified views of tail regions (G’-I’). Whereas the transient knockdown of lgl2 by morpholinos is not able to induce late epidermal defects, F0 Crispr/Cas9 mutants develop epidermal phenotypes starting at 5–6 dpf in about 60% of injected embryos (N = 3, n = 32–40), reminiscent of the lgl2/penner mutant (39, Fig 2B) and demonstrating loss of lgl2 function. (J-L) Brightfield images of live 54 hpf embryos crispant for lgl2 to induce polarity defects, morphant for pax2a to induce hypotonic stress, or both; overviews of entire embryos (J-L) and magnified views of tail regions (J’-L’). Whereas lgl2 knockout by Crispr/Cas9 alone does not result in epidermal defects at early stages, it causes epidermal aggregate formation when combined with the knockdown of pax2a by morpholinos (L,L’; indicated in L’ with arrows) comparable to the knockdown of lgl2 by morpholinos (Fig 7A–7C, 7K), Scale bar: 500 μm in overview, 100 μm in magnified image. (K) Quantification of phenotypes of embryos with different genotypes / morphant conditions (N = 3, n = 27–53). Significances were determined via a one-way ANOVA and Tukey’s post hoc test by comparing fraction of embry [file pgen.1010873.s004.tif]
